# Supplementary material for: Non-coplanar helimagnetism in the layered van-der-Waals metal DyTe3
Source: Nat Commun. 2024 May 20;15:4291. doi: 10.1038/s41467-024-47127-5 (PMC11106302; doi:10.1038/s41467-024-47127-5)
Supplement: Supplementary file 1 — Supplementary Information [file 41467_2024_47127_MOESM1_ESM.pdf]

Non-coplanar helimagnetism in the layered van-der-Waals metal $DyTe_3$

–

Supplementary Information

Shun Akatsuka[1,9], Sebastian Esser[1,9],[*] Shun Okumura[1], Ryota Yambe[1], Rinsuke Yamada[1], Moritz M. Hirschmann[2], Seno Aji[3,8], Jonathan S. White[4], Shang Gao[5], Yoshichika Onuki[2], Taka-hisa Arima[2,6], Taro Nakajima[2,3], and Max Hirschberger[1,2,7†]

[1]*Department of Applied Physics, The University of Tokyo, Bunkyo-ku, Tokyo 113-8656, Japan*

[2]*RIKEN Center for Emergent Matter Science (CEMS), Wako, Saitama 351-0198, Japan*

[3]*The Institute for Solid State Physics, The University of Tokyo, Kashiwa 277-8581, Japan*

[4]*Laboratory for Neutron Scattering and Imaging (LNS), Paul Scherrer Institute (PSI), 5232, Villigen, Switzerland*

[5]*Department of Physics, University of Science and Technology of China, Hefei 230026, China*

[6]*Department of Advanced Materials Science, The University of Tokyo, Kashiwa 277-8561, Japan*

[7]*Quantum-Phase Electronics Center (QPEC), The University of Tokyo, Bunkyo-ku, Tokyo 113-8656, Japan*

[8]*Present address: Department of Physics, Faculty of Mathematics and Natural Sciences, Universitas Indonesia, Depok 16424, Indonesia.* and

[9]*These two authors contributed equally: Shun Akatsuka, Sebastian Esser*

[*] esser@g.ecc.u-tokyo.ac.jp

[†] hirschberger@ap.t.u-tokyo.ac.jp

TABLE I. **Magnetic properties of van-der Waals systems with complex magnetic order.** Electrical transport properties are categorized into metals (M), insulators (I), and materials with metal-to-insulator transition (MIT), and spin textures are classified into coplanar (CP) and noncoplanar (NCP). $DyTe_3$ is the only metallic compound that is (a) incommensurate with the underlying lattice, and (b) has a component of the modulation vector **q** perpendicular to the stacking direction. Moreover, it is a rare example of noncoplanar (NCP) magnetism in a bulk vdW compound.

Compound	Space group	**q**-vector	Transport	Magnetism	Ref.
$DyTe_3$	$Cmcm$	$q_{\rm cyc} \sim (0\ ,\ 1\ ,\ 0.207)$	M	NCP	this work
		$q_{\rm AFM} \sim (0\ ,\ 1\ ,\ 0.5)$			
$Fe_{5-x}GeTe_2$	$R\bar{3}m$	$\pm\frac{1}{3}(1\ ,\ 1\ ,\ 3)$	M	NCP	[1–3]
		$\pm\frac{3}{10}(0\ ,\ 0\ ,\ 3)$			
AgCrSe2		(0.037 , 0.037 , 3/2)	I	CP	[4, 5]
NiI_2		(0.138 , 0 , 1.457)	MIT	CP	[6, 7]
$NiBr_2$		(0.027 , 0.027 , 3/2)	I	CP	[8–10]
CoI_2	$P\bar{3}m1$	(1/12 , 1/12 , 1/2)	-	CP	[6]
		(1/8 , 0 , 1/2)			
MnI_2		(0.181 , 0 , 0.439)	I	CP	[10, 11]
$Co_{1/3}NbS_2$	$P6_322$	(0.5 , 0 , 0)	M	NCP	[12–14]
$Co_{1/3}TaS_2$		(0.5 , 0 , 0)	M	NCP	[15]
$Cr_{1/3}NbS_2$		(0 , 0 , 0.025)	M	NCP	[16–18]
$Cr_{1/3}TaS_2$		(0 , 0 , 0.081)	M	NCP	[16, 19–21]

I. SPIN HAMILTONIAN

Model Hamiltonian. To describe the ground state and the field-induced transition in $DyTe_3$ for $\mathbf{B} \parallel c$, consider a one-dimensional (1D) chain, where each unit cell contains a single magnetic site, of index n, in the paramagnetic state. For this model, the lattice constant is set to $c = 1$, the reciprocal lattice constant is $c^* = 2\pi$, the wavevector q is dimensionless, and the CDW wavenumber (for $DyTe_3$) is $q_{\mathrm{CDW}} = 2\pi \cdot 0.293$ [22]. As compared to the effective zigzag chain in $DyTe_3$, the number of magnetic sites is halved, i.e. the lower part of the chain is omitted. Hence, *ud* order for the component S^a of the local, quasi-classical spin $\mathbf{S}$ is equivalent to *uudd* order on the zigzag chain.

We start with a real-space ansatz comprising antiferromagnetic exchange, a spatially modulated on-site coupling induced by the lattice distortion from the CDW, and a Zeeman term for magnetic field applied along the chain axis (c-axis),

$$\mathcal{H} = \sum_n \left[J_2 S_n^a S_{n+1}^a - E_{\mathrm{CDW}}^{ab} \cos\left(q_{\mathrm{CDW}} z_n\right) S_n^a S_n^b - E_{\mathrm{CDW}}^{ac} \sin\left(q_{\mathrm{CDW}} z_n\right) S_n^a S_n^c + B S_n^c \right] \quad (1)$$

where n labels magnetic moments on a single layer of a single zigzag chain. Here, n, $n+1$ represent nearest neighbours in the crystal lattice of $DyTe_3$, so that their coupling J_2 can be expected to be stronger than the coupling J_1 between the sheets (see next section).

The oscillating off-diagonal terms, $E_{\mathrm{CDW}}^{ab} \cos\left(q_{\mathrm{CDW}} z_n\right)$ and $E_{\mathrm{CDW}}^{ac} \sin\left(q_{\mathrm{CDW}} z_n\right)$ are permitted since the global $\mathcal{M}_b$ and the local $\mathcal{M}_c$-mirror are broken by the lattice distortion due to the CDW, respectively. Moving to Fourier space according to the conventions $\mathbf{S}_n = (1/\sqrt{N}) \sum_q \mathbf{S}_q \exp(iqz_n)$ and $\sum_n \exp(iqz_n) = N\delta(q)$, where N is the number of sites on the chain, we have

$$\mathcal{H} = \sum_q \left[J_2 \cos(q) S_q^a S_{-q}^a - \frac{E_{\mathrm{CDW}}^{ab}}{2} S_q^a \left(S_{-q+q_{\mathrm{CDW}}}^b + S_{-q-q_{\mathrm{CDW}}}^b \right) - \frac{E_{\mathrm{CDW}}^{ac}}{2i} S_q^a \left(S_{-q+q_{\mathrm{CDW}}}^c - S_{-q-q_{\mathrm{CDW}}}^c \right) \right] + B\sqrt{N}\, S_{q=0}^c \quad (2)$$

For a Heisenberg Hamiltonian without off-diagonal terms, the Luttinger-Tisza rule dictates the choice of a single, optimal q for the long-range ordered state; yet here, given the off-diagonal coupling of spin components, we naturally select different wavenumbers for different spin components, explaining the clear separation of spin components by $\mathbf{q}$-vector observed experimentally in Fig. 2 of the main text. Hereafter, we set $E_{\mathrm{CDW}}^{ab} = E_{\mathrm{CDW}}^{ac} = E_{\mathrm{CDW}}$ for simplicity.

If $J_2 > 0$, the minimum of $J_2 \cos(q)$ is at $q = \pi$ and $S^a_{q=\pi}$ is realized. If further $E_{\mathrm{CDW}} > 0$, S^c_q is directly locked to the dominant S^a_q and

$$S^b_q \propto \delta(q = \pi + q_{\mathrm{CDW}}) + \delta(q = \pi - q_{\mathrm{CDW}}) \tag{3}$$

$$S^c_q \propto \delta(q = \pi + q_{\mathrm{CDW}}) - \delta(q = \pi - q_{\mathrm{CDW}}) \tag{4}$$

realizing the coupled wavevectors that are experimentally observed in phase I of $DyTe_3$. Application of a magnetic field B larger than J_2 enforces $S^c_q \propto \delta(q = 0)$ and the cross-terms induce $S^a_q \propto \delta(q = \pm q_{\mathrm{CDW}})$ and the second harmonics $S^b_q \propto \delta(q = \pm 2q_{\mathrm{CDW}})$ demonstrated experimentally in Fig. 5 of the main text. Indeed, the Zeeman energy per magnetic moment of $m_{\mathrm{Dy}} = 10\,\mu_{\mathrm{B}}$ (Bohr magneton) at the critical field $B_{\mathrm{c}} = 0.5\,\mathrm{T}$ in Fig. 5 of the main text is roughly 3.6 K, very close to the value of the Néel temperature T_{N}.

If, in contrast, $E_{\mathrm{CDW}} < 0$, there is no reason why wavenumbers such as $\pi \pm q_{\mathrm{CDW}}$ should appear in the ground state. As compared to anisotropic exchange interactions, for example of the type $S^c_n S^a_{n+1} + S^a_n S^c_{n+1}$, the present E_{CDW} terms cannot induce spontaneous magnetic order by themselves, but rather they create a 'parasitic' spin modulation – driven by the charge-density wave of $R\mathrm{Te}_3$, $R =$rare earth – on the back of either AFM order below B_{c} or of the field-polarized moment above B_{c}.

Additional Heisenberg coupling on zigzag chain. We introduce an additional J_1 between neighbouring sites on the zigzag chain as

$$\mathcal{H}'' = J_1 \sum_n (\mathbf{S}_{n1} \cdot \mathbf{S}_{n2} + \mathbf{S}_{n2} \cdot \mathbf{S}_{n+1,1}) \tag{5}$$

which can be separated into three independent equations $\mathcal{H}''_a$, $\mathcal{H}''_b$, $\mathcal{H}''_c$ – one for each spin component. Note that $\mathbf{S}_{n1}$, $\mathbf{S}_{n+1,1}$ are *not* nearest neighbours in the lattice of $DyTe_3$, so that their coupling J_1 is expected to be weaker than the dominant antiferromagnetic J_2 in Eq. (1). We use the trial functions

$$S^a_{n\alpha} = \bar{S}_a \cos\left(2\pi \cdot \frac{1}{2} \cdot z_{n\alpha} + \varphi^{\mathrm{AFM}}_\alpha\right) \tag{6}$$

$$S^b_{n\alpha} = \bar{S}_b \sin\left(h_\alpha \cdot q_{\mathrm{cyc}} \cdot z_{n\alpha} + \varphi_\alpha\right) \tag{7}$$

$$S^c_{n\alpha} = \bar{S}_c \cos\left(h_\alpha \cdot q_{\mathrm{cyc}} \cdot z_{n\alpha} + \varphi_\alpha\right) \tag{8}$$

with a helicity parameter h_α indicating the propagation *direction* of the texture, the incommensurate wavevector $q_{\mathrm{cyc}} = 2\pi\, L_{\mathrm{cyc}}$ in reciprocal lattice units (r.l.u.), as well as the phases

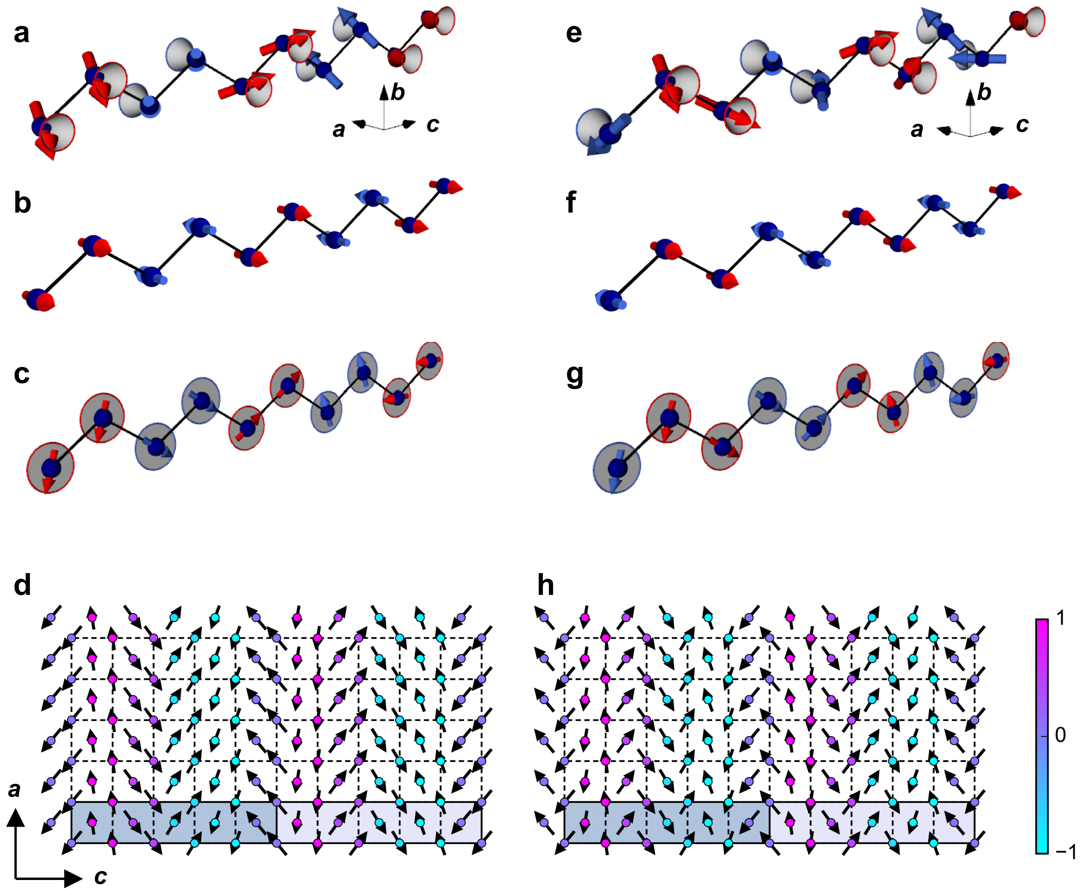

FIG. 1. **Two possible magnetic structures in the ground state of $DyTe_3$.** Neutron scattering does not constrain the combination of cycloidal (S_b, S_c) and antiferromagnetic (AFM, S_a) components, which can be either in-phase (left) or out of phase (right). The AFM and cycloidal components of in-phase (out of phase) combinations are depicted, in panels **b**,**c** (in panels **f**, **g**), respectively. Only a single zigzag chain is shown, although there is antiferromagnetic coupling between subsequent zigzag chains along the b-axis, c.f. Fig. 1 of the main text. **d**,**h** Magnetic texture in a single DyTe magnetic square net bilayer, corresponding to the simplified zigzag-chain picture in panels a,e. Colour on each ionic site (arrows) illustrate the b-axis (the ac-plane) component of the magnetic moments. Dashed lines are guides to the eye, describing the square net in the upper sheet of the DyTe bilayer slab. The bright (dark) highlight marks the size of the magnetic unit cell (the size of half a magnetic unit cell, as shown in panels **a-c**, **e-g**, and in the figures of the main text). For clarity, the relative phase δ between the layers is chosen so that pairs of spins are fully collinear (instead of the experimentally determined 79° phase shift, Section VI).

φ_α and $\varphi_\alpha^{\rm AFM}$. The latter can only vary in steps of π, if we assume a fixed length of the magnetic moment. Based on this,

$$\mathcal{H}''_b = J_1 \bar{S}_b^2 \cos\left(h_1 q_{\rm cyc}/2\right) \sum_n \left[\cos\left((h_2 - h_1) q_{\rm cyc}(n+1/2) + \varphi_2 - \varphi_1\right) - \right.$$
$$\left. - \cos\left((h_2 + h_1) q_{\rm cyc}(n+1/2) + \varphi_2 + \varphi_1\right)\right] \qquad (9)$$
$$\mathcal{H}''_c = J_1 \bar{S}_c^2 \cos\left(h_1 q_{\rm cyc}/2\right) \sum_n \left[\cos\left((h_2 - h_1) q_{\rm cyc}(n+1/2) + \varphi_2 - \varphi_1\right) + \right.$$
$$\left. + \cos\left((h_2 + h_1) q_{\rm cyc}(n+1/2) + \varphi_2 + \varphi_1\right)\right] \qquad (10)$$

and the two cases $p = h_1 \cdot h_2 = +1$ and $p = -1$ yield

$$E''_{p=+1} = J_1\, N \left(\bar{S}_c^2 + \bar{S}_b^2\right) \cos\left(q_{\rm cyc}/2\right) \cos\left(\varphi_2 - \varphi_1\right) \qquad (11)$$
$$E''_{p=-1} = J_1\, N \left(\bar{S}_c^2 - \bar{S}_b^2\right) \cos\left(q_{\rm cyc}/2\right) \cos\left(\varphi_2 + \varphi_1\right) \qquad (12)$$

These terms in Eq. (11) and (12) are independent of z_n; especially for $p = +1$, optimizing $\delta = \varphi_2 - \varphi_1$ yields a favorable energy contribution for any given J_1, $E_{\rm CDW}$.

II. EXPRESSIONS FOR SCATTERING INTENSITIES

We review the expressions used to calculate neutron scattering intensities from atomic and magnetic structures of $DyTe_3$, which serve to define a variety of parameters (such as the phase shift δ) used in the discussion of the main text. Working with the triple-axis diffractometer PONTA-5G at JRR-3 research reactor, we fit ω or $\theta - 2\theta$ scans of neutron intensities with Gaussian profiles and calculate the total observed intensity $I_{\rm obs}$ for each reflection, taking the $\mathbf{Q}$-dependence of the peak shape into account (Fig. 10). From this, we calculate the observed structure factor as

$$F_{\rm obs}(\mathbf{Q}) = \sqrt{I_{\rm obs}(\mathbf{Q})/\mathcal{L}(2\theta)} \qquad (13)$$

where $\mathcal{L}(2\theta) = \lambda^3/\sin(2\theta)$ is the Lorentz factor, λ is the wavelength of the monochromatized neutrons, and 2θ is the scattering angle.

To reproduce this $F_{\rm obs}(\mathbf{Q})$ quantitatively, we start from the expression for the differential cross-sections for nuclear and magnetic scattering, i.e. the beam intensity scattered into a solid angle $\mathrm{d}\Omega$ corresponding to the direction of the momentum transfer $\mathbf{Q}$ of magnitude

$Q = |\mathbf{Q}|$ and direction $\hat{\mathbf{Q}} = \mathbf{Q}/Q$

$$\left(\frac{\mathrm{d}\sigma}{\mathrm{d}\Omega}\right)^N_{\mathrm{cal}} \equiv I^N_{\mathrm{cal}} = \Phi \left| \sum_{j\in\mathrm{lattice}} b_j \exp\left(\imath \mathbf{Q}\cdot\mathbf{r}_j\right)\right|^2 \equiv \Phi \left|F^N_{\mathrm{cal}}(\mathbf{Q})\right|^2 \sum_{\mathbf{l},\mathbf{l}'} \exp\left[\imath \mathbf{Q}\cdot(\mathbf{l}-\mathbf{l}')\right] \quad (14)$$

$$\left(\frac{\mathrm{d}\sigma}{\mathrm{d}\Omega}\right)^M_{\mathrm{cal}} \equiv I^M_{\mathrm{cal}} = \Phi \left| -2.7 \sum_{j\in\mathrm{lattice}} f_{\mathrm{mag}}(Q)\, \mathbf{m}_{\perp,j} \exp\left(\imath \mathbf{Q}\cdot\mathbf{r}_j\right)\right|^2 \quad (15)$$

Here, we have introduced the neutron flux Φ, the position of each atom on the lattice $\mathbf{r}_j$, the nuclear scattering length b_j, the unit cell coordinates $\mathbf{l}$, $\mathbf{l}'$, the magnetic form factor $f_{\mathrm{mag}}(Q)$ evaluated from an analytic expression [23], and the component of the magnetic moment at site j that is perpendicular to the momentum transfer

$$\mathbf{m}_{\perp,j}(\mathbf{Q}) = \mathbf{m}_j - \left(\mathbf{m}_j \cdot \hat{\mathbf{Q}}\right)\cdot \hat{\mathbf{Q}} \quad (16)$$

Note the scattering lengths for Dy^{3+} (16.9 fm) and Te (5.8 fm), and that only magnetic ions contribute to Eq. (15).

For nuclear scattering, we use according to Ref. [24]

$$F^N_{\mathrm{cal}}(\mathbf{Q}) = \sum_{\mathbf{d}\in\mathrm{c.u.c.}} b_j \exp\left(\imath\, \mathbf{Q}\cdot\mathbf{d}\right) \quad (17)$$

$$\Phi \sum_{\mathbf{l},\mathbf{l}'} \exp\left[\imath \mathbf{Q}\cdot(\mathbf{l}-\mathbf{l}')\right] = \Phi\, N \frac{(2\pi)^3}{\nu_0}\, \delta^{(3)}(\mathbf{Q}-\mathbf{G}) \quad (18)$$

for a reciprocal lattice vector $\mathbf{G}$ and using $\mathbf{r}_j = \mathbf{d} + \mathbf{l}$, where the former is a coordinate within the crystallographic unit cell, and the latter labels the origin of each unit cell. Φ, N, F^N_{cal}, are the flux of incident neutrons, the number of crystallographic unit cells (c.u.c., of volume ν_0) in the sample, and the nuclear structure factor. We find good agreement of the experimental scattering data and model when using the atomic positions from the high-temperature *Cmcm* space group of $DyTe_3$ [25]. In reality, the formation of charge order below $T_{\mathrm{CDW}} \approx 320$ K lowers the symmetry, as discussed by Malliakas *et al.* in Refs. [22, 26].

Then, a scale factor s is defined by equating to the experimentally observed intensity,

$$\left|F^N_{\mathrm{obs}}(\mathbf{Q})\right|^2 = \Phi\, N\, \frac{(2\pi)^3}{\nu_0} \left|F^N_{\mathrm{cal}}(\mathbf{Q})\right|^2 \equiv s \left|F^N_{\mathrm{cal}}(\mathbf{Q})\right|^2 \quad (19)$$

as shown in Fig. 7.

Next, for magnetic scattering from a structure with lattice-commensurate magnetic order,

we use Eq. (18) with a larger unit cell (reduced set of $\mathbf{G}$) and equate

$$\left|F^M_{\text{obs}}(\mathbf{Q})\right|^2 = I^M_{\text{obs}}(\mathbf{Q})/\mathcal{L}(2\theta) = I^M_{\text{cal}}(\mathbf{Q}) = \Phi\, N_M\, \frac{(2\pi)^3}{\nu_M} \left|\mathbf{F}^M_{\text{cal}}(\mathbf{Q})\right|^2 \equiv s \left|\frac{\mathbf{F}^M_{\text{cal}}}{x}\right|^2 \tag{20}$$

$$\mathbf{F}^M_{\text{cal}}(\mathbf{Q}) = -2.7 f_{\text{mag}}(Q) \sum_{j\in\text{m.u.c.}} \mathbf{m}_{\perp,j}\, \exp(\imath\mathbf{Q}\cdot\mathbf{r}_j) \tag{21}$$

We introduced the calculated magnetic structure factor $\mathbf{F}^M_{\text{cal}}$, which has three complex components $F^M_{\text{cal},x}$, $F^M_{\text{cal},y}$, $F^M_{\text{cal},z}$; the volume of the magnetic unit cell (m.u.c.) $\nu_M = x \cdot \nu_0$, the magnetic form factor of Dy^{3+}, f_{mag}, and the number $N_M = N/x$ of m.u.c. in the sample. The prefactor (-2.7) describes the scattering length of the electron, and the sum is now over all magnetic (dysprosium) ions in the m.u.c.

In case of two domains of this commensurate order, as relevant for the analysis in $DyTe_3$, we have

$$I^M_{\text{cal}} = \Phi\, \frac{N}{2}\, \frac{(2\pi)^3}{\nu_0} \left|\frac{\mathbf{F}^{M1}_{\text{cal}}}{x}\right|^2 + \Phi\, \frac{N}{2}\, \frac{(2\pi)^3}{\nu_0} \left|\frac{\mathbf{F}^{M2}_{\text{cal}}}{x}\right|^2 = \frac{s}{2}\left(\left|\frac{\mathbf{F}^{M1}_{\text{cal}}}{x}\right|^2 + \left|\frac{\mathbf{F}^{M2}_{\text{cal}}}{x}\right|^2\right) \tag{22}$$

where $\mathbf{F}^{Mk}_{\text{cal}}$, $k = 1, 2$ are the magnetic structure factors of the two domains, respectively.

III. SYMMETRY AND STRUCTURE FACTOR: COMMENSURATE COMPONENT IN PHASE I

A. Symmetry consideration (commensurate)

Section I discusses oscillatory terms that are allowed in the Hamiltonian due to local symmetry breaking from the charge-density wave (CDW). In this section, we focus on *global* symmetries of $DyTe_3$ and their lowering by magnetic order. We start from space group $Cmcm$, ignoring the incommensurate CDW at first, and briefly consider the effect of the CDW on global symmetries at the end of the section.

In polarized neutron scattering (PNS), orthorhombic symmetry of the crystal structure allows us to set the scattering plane to $(HK0)$, with separation of three orthogonal magnetization components m_a, m_b, and m_c. The PNS data strictly constrains the commensurate moment to be along the a-axis, and the observation of reflections of the type $(0, \text{odd}, 0.5)$ – while $(0, \text{even}, 0.5)$ are absent –, establishes a unit cell with eight magnetic dysprosium ions, vanishing net magnetization along the a-axis, as well as opposite directions for moments

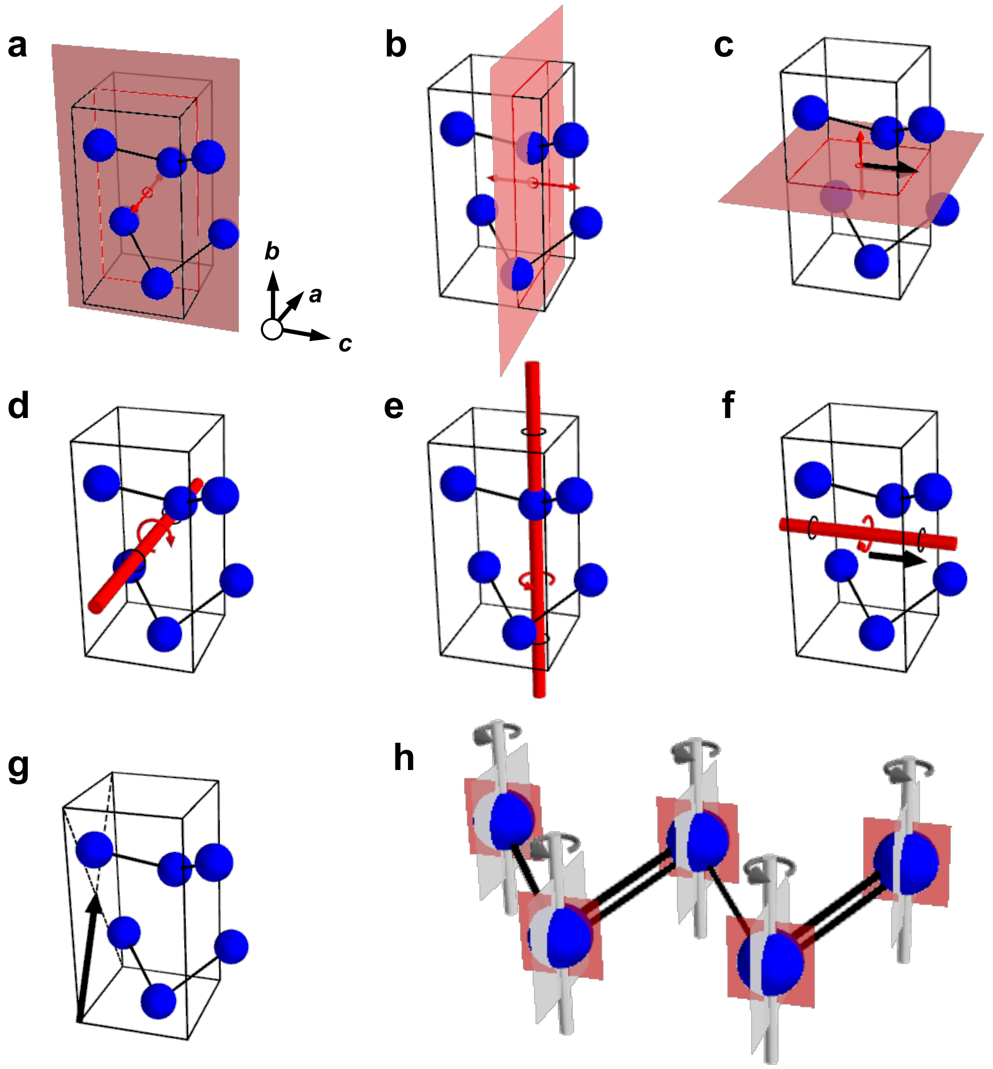

FIG. 2. **Space group** $Cmcm$ **and its symmetries in the** $(000)+$ **set, as well as breaking of bond-symmetry by AFM commensurate order.** Only dysprosium ions are shown (blue), including two ions outside the crystallographic unit cell (right) to illustrate the structural zigzag chain feature. **a**, Mirror plane $\mathcal{M}_a$. **b**, Mirror plane $\mathcal{M}_c$. **c**, Glide mirror plane $\mathcal{G}_b$ with $c/2$ translation along the c-axis (black arrow). **d**, Two-fold rotation C_{2a} through the inversion center $\mathcal{I} = (\frac{1}{2}, \frac{1}{2}, \frac{1}{2})$. **e**, Two-fold rotation C_{2b} through a position shifted by $\left(0, 0, \frac{1}{4}\right)$ from $\mathcal{I}$. **f**, Screw rotation $C_{2c} \times \left(0, 0, \frac{1}{2}\right)$ with $c/2$ shift along the c-axis. **g**, Base centering operation C, i.e., a translation by $\left(\frac{1}{2}, \frac{1}{2}, 0\right)$. **h**, In the presence of commensurate order $\mathbf{q}_{\mathrm{AFM}} = (0, b^*, 0.5c^*)$, the magnetic space group symmetry is lowered to C_c2/m: $\mathcal{M}_c$ and C_{2b} are broken (grey), while $\mathcal{M}_a$ survives (red). The equivalence of bonds on the effective zigzag chain (main text) is lifted in presence of *uudd* or *uddu* order (black double and single bonds).

separated by a distance $b/2$ along the b-axis (Fig. 3c). Four free parameters remain, namely the lengths of four magnetic moments m_a in a DyTe slab spanning two crystallographic unit cells, i.e. the upper zigzag chain in Fig. 3.

The full Hermann-Mauguin symbol for orthorhombic space group $Cmcm$ (SG63) is $C\ 2/m\ 2/c\ 2_1/m$, and $\mathbf{q}_{\mathrm{AFM}} = (0, 1, 0.5)$ does not break any of these symmetries. Using the **k**-Subgroupsmag tool of the Bilbao crystallographic server [27], we determined the highest-symmetry magnetic subgroups of $Cmcm$ that are consistent with $\mathbf{q}_{\mathrm{AFM}}$. In (black-and-white-type) Belov-Neronova-Smirnova notation: I_ama2, C_c2/c, and C_c2/m. From the transformation matrices provided by this tool, we also find the lattice vectors $\mathbf{a}'$, $\mathbf{b}'$, $\mathbf{c}'$ in terms of the lattice vectors $\mathbf{a}$, $\mathbf{b}$, $\mathbf{c}$ of the 'parent' $Cmcm$: $(-2\mathbf{c}, \mathbf{a}, -\mathbf{b})$, $(-\mathbf{b} - 2\mathbf{c}, \mathbf{a}, 2\mathbf{c})$, and $(\mathbf{b} + 2\mathbf{c}, -\mathbf{a}, 2\mathbf{c})$, for the three abovementioned symbols. Here, for example, $\mathbf{b} = \mathbf{e}_b \cdot b$ for basis (unit) vector $\mathbf{e}_b$ and lattice constant b (and so on).

We rule out I_ama2 and C_c2/c for the commensurate order in $DyTe_3$. The former has a $\mathcal{M}_{a'}$ mirror plane, which is perpendicular to $\mathbf{e}_c$ of the $Cmcm$ cell and located on the Dy-sites. A moment m_a on the Dy-site is inconsistent with this mirror plane. The latter space group has a glide mirror plane $\mathcal{G}_{b'}$, consisting of a mirror operation perpendicular to $\mathbf{e}_a$ combined with a lattice translation by $\mathbf{c}$. The moment direction m_a at a given site is unchanged under $\mathcal{G}_{b'}$, but translated by half the length of the magnetic unit cell. Given there are merely four sites in the upper DyTe slab of the magnetic unit cell, this operation requires orders of the type udud, and is thus inconsistent with the expansion of the unit cell along $\mathbf{e}_c$ that is implied by $\mathbf{q}_{\mathrm{AFM}}$.

We focus on monoclinic, centrosymmetric C_c2/m, where C_c includes base centering as a translation $\mathcal{T}_1 = (1/2, 1/2, 0)$ and $\mathcal{T}_2'$, i.e. the translation $\mathcal{T}_2 = (0, 0, 1/2)$ combined with time reversal. There are two domains A and B with basis vectors $(\mathbf{b} + 2\mathbf{c}, -\mathbf{a}, 2\mathbf{c})$ and $(-\mathbf{b} + 2\mathbf{c}, \mathbf{a}, 2\mathbf{c})$; i.e., they are characterized by a reversal of the monoclinic tilt (Fig. 3). Only the mirror plane perpendicular to $\mathbf{e}_a$ remains intact, the number of inversion centers is halfed as compared to $Cmcm$, and the broken c-mirror relates the two possible domains A and B depicted in Fig. 3 **c**.

Note: A previous x-ray scattering study reports the superspace group $C2cm(00\gamma)000$ for the CDW state in $DyTe_3$ [26]. In average space group $C2cm$ (No. 40), the $\mathcal{M}_a$ mirror of $Cmcm$ is already broken. Starting from this lower-symmetry symbol, analogous discussion leads to C_c2 for the commensurate component of the magnetic order in phase I.

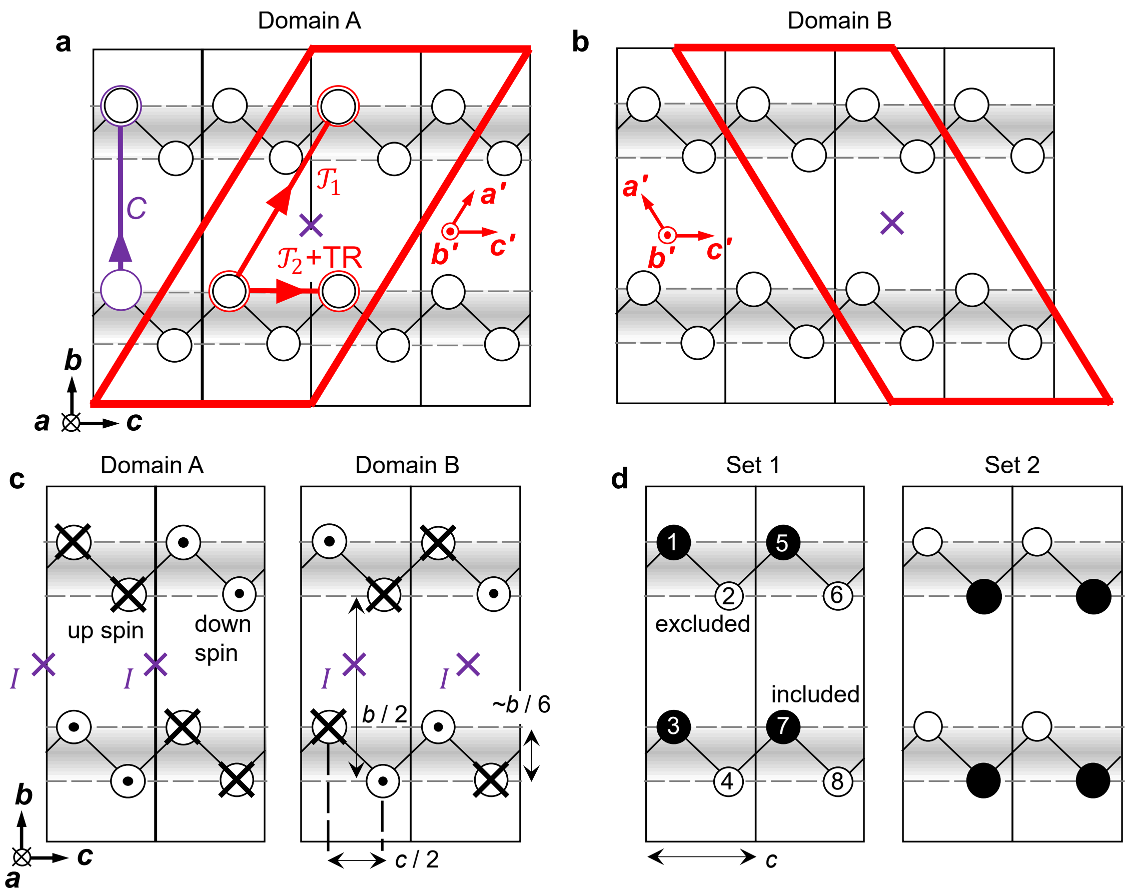

FIG. 3. **Unit cell, magnetic domains for commensurate spin component, and illustration for structure factor calculation in phase I of $DyTe_3$ (antiferromagnetic part, AFM).** **a**, **b**, Primitive magnetic unit cell (red boundary, monoclinic) in two AFM domains of $DyTe_3$, with base centering translation vector $\mathcal{T}_1$ and C_c translation vector (plus time reversal) $\mathcal{T}_2$. The base centering operation of the parent structure $Cmcm$ (C), and its inversion center (I), are also indicated by a violet arrow and cross, respectively. Only magnetic sites are shown, and sites with different x-positions have been projected onto a single plane. See Section III. **c**, Conventional unit cell for two AFM domains in phase I. Circles with dot (with cross) signify magnetic moment pointing into (out of) the plane of the figure. The b-axis spacing of magnetic sites in a bilayer (grey shading) is approximately $b/6$. Inversion centers $\mathcal{I}$ are indicated by violet crosses. **d**, Two sets of sites (black, white circles) of magnetic ions in the unit cell. In the calculation of the structure factor for the combined scattering of two equally populated domains A, B according to Eq. (23), the only contributions are from terms $\sim \exp(\imath \mathbf{Q} \cdot (\mathbf{r}_i - \mathbf{r}_j))$ for sites i, j in the same set.

We consider the possibility of further symmetry lowering, as caused by non-uniform magnetic moment length m_a. Magnetic space group C_c2/m has $\mathcal{T}_2'$, which ensures that moments m_a in the same layer α point in opposite directions, but have the same length. Instead of *uudd* (up-up-down-down), this leaves $(u_{\text{short}}u_{\text{long}}d_{\text{short}}d_{\text{long}})$ as a viable configuration, but C_{2a} rotation symmetry interrelates layers $\alpha = 1, 4$ and eliminates this possibility. The neutron data are well described by C_c2/m (or C_c2, which leads to the same commensurate structure models), so that a further symmetry lowering – relaxing the uniformity constraint on m_a – is deemed unnecessary.

B. Structure factor calculation (commensurate)

We now derive explicit expressions for the scattered intensity at commensurate (AFM) reflections in momentum space, in phase I of $DyTe_3$.

For momentum transfer $\mathbf{Q}$ in the $(0KL)$ plane, we define a normalized moment for each domain $\mathbf{m}_{\perp,j} = m_a\, f_{A,B}(\mathbf{r}_j)\, \mathbf{e}_a$, where $f_{A,B} = \pm 1$. Assuming equal population of domains A and B, Eq. (22) gives

$$I^M_{\text{cal}} = \frac{s}{8}\left(-2.7\, f_{\text{mag}}(Q)\, m_a\right)^2 \cdot \sum_{i,j=1,\ldots,8} \exp\left[\imath \mathbf{Q}\cdot(\mathbf{r}_i - \mathbf{r}_j)\right] \cdot \left[f_A(\mathbf{r}_i) f_A^*(\mathbf{r}_j) + f_B(\mathbf{r}_i) f_B^*(\mathbf{r}_j)\right] \tag{23}$$

where the magnetic sites i, j are labeled in Fig. 3 **d** (not Fig. 2 of the main text). In the latter edgy brackets, the two terms cancel (sum to ± 2) when the relationship between magnetic moments at site i, j is opposite (the same) in domains A, B. In addition to four pairs of type $i = j$, examination of Fig. 3 **d** shows that, for the purpose of evaluating Eq. (23), the magnetic sites can be split into two sets 1 and 2: the non-vanishing terms in the sum correspond to pairs of magnetic moments that are just above / below one another, or two sites apart along the c-direction.

The relative distances between sites are the same for set 1 and 2, and there are three types of site pairs i, j in each set:

$$\mathbf{r}_i - \mathbf{r}_j = \pm c\, \mathbf{e}_c \tag{24}$$

$$\mathbf{r}_i - \mathbf{r}_j = \pm (b/2)\, \mathbf{e}_b \tag{25}$$

$$\mathbf{r}_i - \mathbf{r}_j = \pm (c\, \mathbf{e}_c - (b/2)\, \mathbf{e}_b) \tag{26}$$

with both $\pm$ appearing in the sum of Eq. (23). The moments are antiparallel for all pairs of sites, $[f_A(\mathbf{r}_i)f_A^*(\mathbf{r}_j) + f_B(\mathbf{r}_i)f_B^*(\mathbf{r}_j)] = -2$, except the pairs in Eq. (26). We specialize to the $(0, Kb^*, Lc^* + q_{\mathrm{AFM}})$ scattering plane, so that

$$\mathbf{Q} = K\frac{2\pi}{b}\,\mathbf{e}_b + (L+0.5)\frac{2\pi}{c}\,\mathbf{e}_c \tag{27}$$

as in our experiment, and obtain

$$\begin{aligned}\sum_{i\neq j,set1} \exp(\imath\mathbf{Q}\cdot(\mathbf{r}_i-\mathbf{r}_j)) &= 4\cos(Q_c\,c) + 4\cos\left(Q_b\,\frac{b}{2}\right) - 4\cos(Q_c\,c)\cos\left(Q_b\frac{b}{2}\right)\\ &= -4 + 4\cdot(-1)^K + 4\cdot(-1)^K = 4\left(2(-1)^K - 1\right)\end{aligned} \tag{28}$$

which takes the values -12 $(+4)$ for $K =$odd (even), independent of L. Then, $\left|F^M_{\mathrm{obs}}\right| \equiv 0$ when K is even, and for $K =$odd,

$$\begin{aligned}I^M_{\mathrm{cal}} &= \frac{s}{8}\left(-2.7 f_{\mathrm{mag}}(Q)\, m_a\right)^2 \left(2\cdot 8 - 2\cdot 2\cdot(-12)\right)\\ &= 8s\left(-2.7 f_{\mathrm{mag}}(Q)\, m_a\right)^2\end{aligned} \tag{29}$$

without any explicit dependence on K and L both (but note the Q dependence of the magnetic form factor). The above discussion shows that contributions from domains A, B partially cancel each other, meaning that the ratio of domains can be refined from the data.

IV. SYMMETRY AND STRUCTURE FACTOR: INCOMMENSURATE COMPONENT IN PHASE I

A. Symmetry consideration (incommensurate)

Starting from Eqs. (7) and (8), and given antiferromagnetic coupling between structural bilayers in $DyTe_3$, as enforced by $K =$odd in $(0, K, L_{\mathrm{cyc}})$, the phase relation between layers $\alpha = 1, 3$ and $2, 4$ is fixed to π and $h_1 = h_3$, $h_2 = h_4$. The remaining free parameters, besides $\bar{S}_b$, $\bar{S}_c$, are a single phase $\delta = \varphi_1 - \varphi_2 = \varphi_3 - \varphi_4$ and the parameter $p = h_1 \cdot h_2 = \pm 1$, which characterizes the relative helicity in two adjacent layers. We set a single (possibly distorted), incommensurate cylcoid into the uppermost layer $\alpha = 1$ with $\varphi_1 = 0$, and – enforced by $K =$odd – a copy with $\phi_3 = \pi$ into the third layer. We then approximate the incommensurate order by an (arbitrarily large) supercell, and discuss the symmetries that leave the magnetic moments unchanged. From this, we deduce constraints on p and δ.

Starting from C_c2/m, the cycloid in $\alpha = 1$ removes the $\mathcal{M}_a$ mirror operation. Figure 4 **a** shows the point group symmetries of a cycloid, with one of the m' operations perpendicular to $\mathbf{e}_a$ in $DyTe_3$; but the (mirror $\times$ time reversal) operation is not consistent with the commensurate component of phase I. Instead, a c-glide appears in the place of $\mathcal{M}_a$ (in the frame of $Cmcm$). The translations $\mathcal{T}_1$ and $\mathcal{T}_2'$ of C_c (Fig. 3) are broken by the cy-

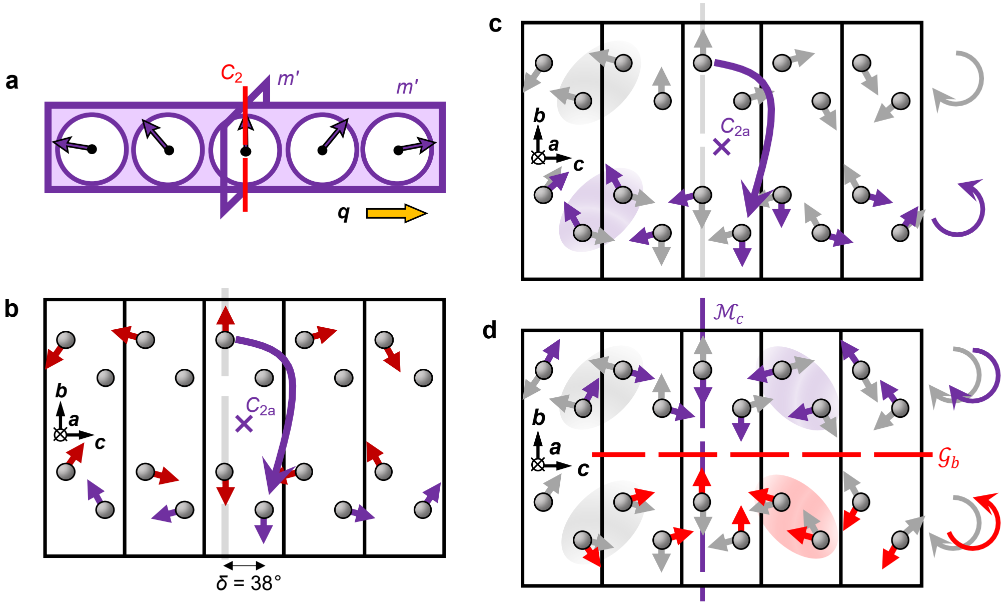

FIG. 4. **Symmetry considerations regarding incommensurate magnetic order in phase I, for $DyTe_3$.** **a**, Magnetic point group symmetries of a cycloid. **b**, A magnetic texture with $P2$ symmetry and ordering vector $\mathbf{q}_{\rm cyc}$. Red arrows: Setting a cycloid in the uppermost layer $\alpha = 1$ with helicity $h_1 = +1$, the $\alpha = 3$ layer's structure with $h_3 = +1$ is imposed by antiferromagnetic coupling ($K = 1$). Violet arrows: Applying C_{2a} symmetry enforces a counter-propagating cycloid in layers $\alpha = 2, 4$, with $h_2 = h_4 = -1$, where the phase $\delta = \varphi_2 - \varphi_1$ is fixed to 38°. The texture in layer $\alpha = 2$ can be inferred from AFM coupling to $\alpha = 4$. **c**, Switching between helicity domains in the Cc model with uniform helicity $\mathbf{h} = \pm(1, 1, 1, 1)$; for simplicity of the illustration, the phase difference is set to $\delta = 38\,°$. Grey arrows: assuming broken C_{2a} symmetry, uniform helicity and adjustable phase shift δ are allowed. The C_{2a} operation (violet arrow) maps $\mathbf{h} \leftrightarrow -\mathbf{h}$, while leaving δ unchanged. (Nearly) collinear blocks, (purple highlight) remain coupled. **d**, Simultaneous switching of δ and $\mathbf{h}$ via glide mirror $\mathcal{G}_b$, and switching of δ without $\mathbf{h}$ by mirror $\mathcal{M}_c$. Highlighted ovals mark pairs of spins with nearly collinear alignment in the bc-plane. Again, $\delta = \pm 38$ is used for the purpose of this illustration, although the experimental value is 79°.

cloids in $\alpha = 1, 3$. Without further symmetry lowering, the average magnetic space group is $C2/c$, where – somewhere along the c-direction – a global inversion center survives. Due to the remaining C_{2a} symmetry, the four layers are stacked as $\mathbf{h}_a = (1, -1, 1, -1)$, where $\mathbf{h} = (h_1, h_2, h_3, h_4)$; recall that $h_1 = h_3$ and $h_2 = h_4$ (Fig. 4**b**). Importantly, the configuration $\mathbf{h}_b = (-1, 1, -1, 1)$ is not related to $\mathbf{h}_a$ by a broken symmetry and does not have the same energy as $\mathbf{h}_a$; the energy difference between these two is largely determined by the local Dzyaloshinskii-Moriya (local DMI) interaction on the J_2 bond of the zigzag chain (Fig. 1 of the main text), i.e. the local DMI between nearest neighbors on a single square net of the DyTe slab. The competition between inter-layer Heisenberg exchange J_1, Eq. (5), and intra-layer local DMI determines the choice of co-propagating or counter-propagating cycloids, and hence the average magnetic space group.

For the $\mathbf{h}_a = (1, -1, 1, -1)$ stack, a degree of freedom remains regarding the phase relation

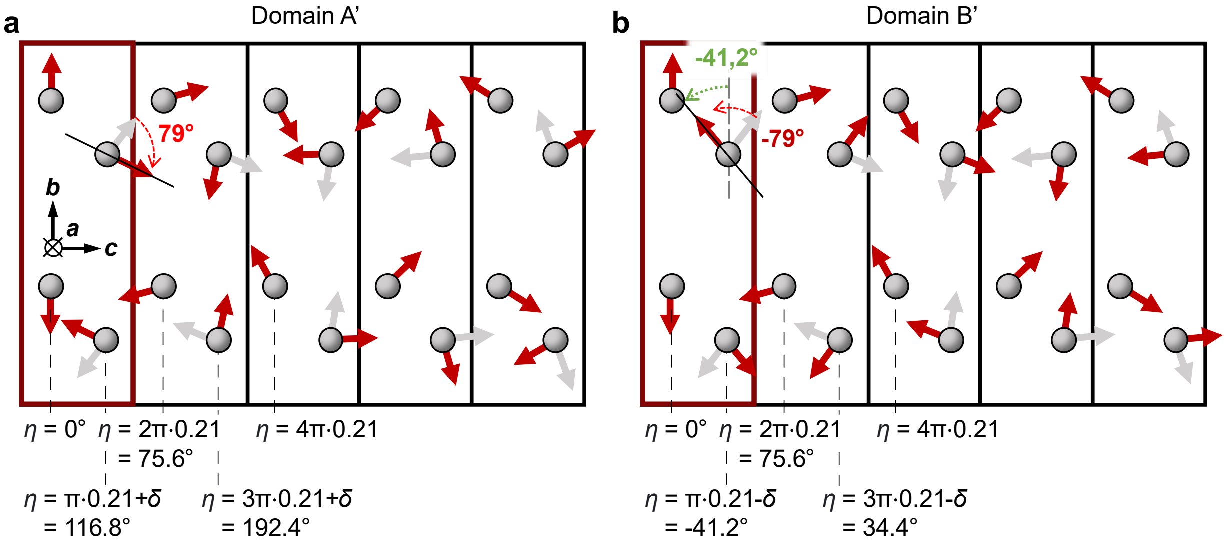

FIG. 5. **Two 'δ-domains' (A$'$ and B$'$), consistent with the $\mathbf{q}_{\mathrm{AFM}} = (0, b^*, 0.5c^*)$ ordering vector, for the incommensurate part of the magnetic order in phase I of $DyTe_3$.** The value of the phase shift $\delta = 79°$ between sheets in a bilayer is adjusted to minimize the reliability index R for the structure factor F. Grey arrows indicate the original moment direction that is expected without any phase shift δ. Note that there is a phase shift of π between upper and lower Dy-bilayer, and that reversing the sign of the helicity vector $\mathbf{h} = (h_1, h_2, h_3, h_4)$, as defined in Section IV, does not affect the value of scattering intensities; η is the angle between the bc-component of the magnetic moment and the crystallographic b-axis.

of cycloids in two layers of a DyTe slab. In particular, the phase shift angle δ determines whether blocks of parallel moments point along $\mathbf{e}_b$ and blocks of antiparallel moments point along $\mathbf{e}_c$, or vice versa. Absent further symmetry lowering, the remaining C_{2a} symmetry in $C2/c$ fixes $h_4 = -h_1$, with $\delta_0 = \pm q_{\rm cyc} \cdot 0.5c \approx \pm 0.207\pi = \pm 37.8°$, where the sign depends on the position of the inversion center in C_c2/m, i.e., on domain A or B of the AFM order. We have derived an analytic expression for the magnetic structure factor of counter-propagating cycloids in $DyTe_3$, Section IV D, and consider the alternative models of high-symmetry, counter-propagating cycloids in that section.

For $\mathbf{h} = (1, -1, 1, -1)$, we can further lower symmetry to Cc (on average, space group number 9) by shifting δ away from δ_0, thus breaking C_{2a}. However, the spontaneous formation of helimagnetism with a unique sense of rotation is common in zigzag chain magnets, and more generally in systems where two or more sublattices are connected by space inversion, a twofold screw axis along the chain, and / or a glide mirror. Examples are $Ni_3V_2O_8$ [28], CuO [29], and the zigzag chain magnet $MnWO_4$ [30], which all host single-sense helices under these conditions. Thus, we consider the cycloid of uniform helicity $\mathbf{h}^{(1)} = (1, 1, 1, 1)$ (average space group Cc), which allows the spin system to adjust δ to minimize inter-chain exchange energy (Section I). We are left with two helicity-domains, $\mathbf{h}^{(1)}$ and $\mathbf{h}^{(2)} = -\mathbf{h}^{(1)}$ for each AFM domain (*uudd* or *uddu*). These are related by C_{2a}, which reverses the rotation sense of the cycloids, but maintains the same AFM domain and the same phase relation δ for a given bond (Fig. 4 **c**). As the symmetry is reduced to Cc in each domain, there is no constraint on the number value of δ. The discussion remains qualitatively unchanged if the commensurate part has magnetic space group C_c2.

Figure 2 **h** demonstrates the inequivalence of bonds on the zigzag chain, caused by the commensurate magnetic structure component, which is essential for symmetry reduction and for allowing off-diagonal anisotropy terms such as $S_n^a S_n^c$ in the Hamiltonian of Section I. In particular, δ becomes a refinable parameter only when the *average* symmetry is lowered to Cc.

B. Structure factor calculation (incommensurate)

We translate the the spin-chain model, Eqs. (7) and (8), into expressions suitable for structure factor calculations. Instead of the full, three-dimensional propagation vector $\mathbf{q}_{\rm cyc} =$

$(0, b^*, 0.207\, c^*)$, we use $\mathbf{q}'_{\rm cyc} = (0, 0, 0.207\, c^*)$, which helps to define the equations and relative phases of magnetic layers in language that is consistent with the main text and with Section IV A. The magnetic sites are labeled by index j as in Fig. 2 of the main text, $\mathbf{r}_j = \mathbf{l} + \mathbf{d}$, where $\mathbf{l}$ and $\mathbf{d}$ label the origin of the unit cell and the intra-cell coordinate of the site, respectively. As there is only one magnetic (dysprosium) site per layer α [defined in Eqs. (7) and (8)], we replace the layer index α by d and write

$$\begin{aligned} \mathbf{m}_j &= 2\mathbf{X} \cos\left[h_d \cdot \mathbf{q}'_{\rm cyc} \cdot (\mathbf{d} + \mathbf{l}) + \varphi_d\right] - 2\mathbf{Y} \sin\left[h_d \cdot \mathbf{q}'_{\rm cyc} \cdot (\mathbf{d} + \mathbf{l}) + \varphi_d\right] \\ &= (\mathbf{X} + \imath\, \mathbf{Y}) \exp\left(\imath\, h_d \cdot \mathbf{q}'_{\rm cyc} \cdot (\mathbf{d} + \mathbf{l}) + \imath\varphi_d\right) + \\ &+ (\mathbf{X} - \imath\, \mathbf{Y}) \exp\left(-\imath\, h_d \cdot \mathbf{q}'_{\rm cyc} \cdot (\mathbf{d} + \mathbf{l}) - \imath\varphi_d\right) \end{aligned} \tag{30}$$

where $\mathbf{X} = X\, \mathbf{e}_X$, $\mathbf{Y} = Y\, \mathbf{e}_Y$ with two orthogonal unit vectors $\mathbf{e}_X$, $\mathbf{e}_Y$. As before, the phase degree of freedom φ_d is specific to each layer index $d(\mathbf{r}_j) = 1, 2, 3, 4$, and the prefactors $h_d = \pm 1$ indicate the helicity (sense of rotation, or handedness) in a layer. Based on the discussion in Section IV A, we use $\varphi_1 = 0$, $\varphi_2 = \pm\delta$, $\varphi_3 = \pi$, and $\varphi_4 = \pi \pm \delta$, where $\pm$ indicates two magnetic domains of the phase shift angle δ.

We insert this into Eq. (15), drop the helicity factor h_d, and define $\mathbf{X}_\perp = \mathbf{X} - \mathbf{X} \cdot \hat{\mathbf{Q}}$ with a normal vector $\hat{\mathbf{Q}} = \mathbf{Q}/\left|\mathbf{Q}\right|$. The quantity $\mathbf{Y}_\perp$ is defined analogously, and we are careful to consider the structure factor in the incommensurate case as a sum over the full lattice, not over a single magnetic unit cell,

$$\begin{aligned} I^M_{\rm cal} &= \Phi \left|-2.7\, f_{\rm mag}(Q)\right|^2 \left(\mathbf{X}^2_\perp + \mathbf{Y}^2_\perp\right) \cdot \\ &\cdot \left[\left|\tilde{F}^+(\mathbf{Q})\right|^2 \sum_{\mathbf{l},\mathbf{l}'} \exp\left(\imath(\mathbf{Q} + \mathbf{q}'_{\rm cyc}) \cdot (\mathbf{l} - \mathbf{l}')\right) + \left|\tilde{F}^-(\mathbf{Q})\right|^2 \sum_{\mathbf{l},\mathbf{l}'} \exp\left(\imath(\mathbf{Q} - \mathbf{q}'_{\rm cyc}) \cdot (\mathbf{l} - \mathbf{l}')\right)\right] \end{aligned} \tag{31}$$

$$\tilde{F}^+(\mathbf{Q}) = \sum_{\mathbf{d} \in \text{c.u.c.}} \exp\left(\imath(\mathbf{Q} + \mathbf{q}'_{\rm cyc}) \cdot \mathbf{d} + \imath\varphi_d\right) \tag{32}$$

$$\tilde{F}^-(\mathbf{Q}) = \sum_{\mathbf{d} \in \text{c.u.c.}} \exp\left(\imath(\mathbf{Q} - \mathbf{q}'_{\rm cyc}) \cdot \mathbf{d} - \imath\varphi_d\right) \tag{33}$$

Now, the sums in Eq. (32) and (33) are over a single chemical unit cell (c.u.c.). This can be rewritten, due to the periodicity of the lattice, as

$$\begin{aligned} I^M_{\rm cal} &= \Phi \left|-2.7\, f_{\rm mag}(Q)\right|^2 \left(\mathbf{X}^2_\perp + \mathbf{Y}^2_\perp\right) \left(N \frac{(2\pi)^3}{\nu_0}\right) \cdot \\ &\cdot \left[\left|\tilde{F}^+(\mathbf{Q})\right|^2 \sum_{\mathbf{G}} \delta(\mathbf{Q} + \mathbf{q}'_{\rm cyc} - \mathbf{G}) + \left|\tilde{F}^-(\mathbf{Q})\right|^2 \sum_{\mathbf{G}} \delta(\mathbf{Q} - \mathbf{q}'_{\rm cyc} - \mathbf{G})\right] \end{aligned} \tag{34}$$

where $\mathbf{G}$ are the reciprocal lattice vectors. Enforced by the δ-functions, the $\tilde{F}^{\pm}$ depend only on the reciprocal lattice vector $\mathbf{G}_0(\mathbf{Q})$ from which the incommensurate reflection 'originates', i.e. $\mathbf{Q} = \mathbf{G}_0 + \mathbf{q}'_{\mathrm{cyc}}$ and $\tilde{F}^{\pm}(\mathbf{Q}) \equiv \tilde{F}^{\pm}(\mathbf{G}_0)$, with

$$\mathbf{G}_0 = H_0 \frac{2\pi}{a}\,\mathbf{e}_a + K_0 \frac{2\pi}{b}\,\mathbf{e}_b + L_0 \frac{2\pi}{c}\,\mathbf{e}_c \tag{35}$$

and H_0, K_0, L_0 integers.

Specializing to the $(0KL)$ scattering plane, $\tilde{F}^{\pm}$ can be calculated explicitly by considering four types of pairs $\mathbf{d} = \mathbf{d}'$, as well as four types of non-identical partners (we omit the a-component of each vector),

$$\mathbf{d}_1 - \mathbf{d}_3 = \mathbf{d}_2 - \mathbf{d}_4 = \frac{b}{2}\,\mathbf{e}_b, \qquad \Delta\varphi_{1,3} = -\pi \tag{36}$$

$$\mathbf{d}_2 - \mathbf{d}_1 = \mathbf{d}_4 - \mathbf{d}_3 = \frac{c}{2}\,\mathbf{e}_c - b_0\,\mathbf{e}_b, \qquad \Delta\varphi_{2,1} = \delta \tag{37}$$

$$\mathbf{d}_4 - \mathbf{d}_1 = \frac{c}{2}\,\mathbf{e}_c - \left(\frac{b}{2} + b_0\right)\,\mathbf{e}_b, \qquad \Delta\varphi_{4,1} = \delta + \pi \tag{38}$$

$$\mathbf{d}_2 - \mathbf{d}_3 = \frac{c}{2}\,\mathbf{e}_c + \left(\frac{b}{2} - b_0\right)\,\mathbf{e}_b, \qquad \Delta\varphi_{2,3} = \delta - \pi \tag{39}$$

with b_0 the spacing, close to $b/6$, between two layers in a covalently bonded bilayer (Fig. 3). Due to the above definition of $\mathbf{q}'_{\mathrm{cyc}}$, these phases are identical for the calculation of both $\tilde{F}^{\pm}(\mathbf{G}_0)$. The parameter δ by itself is either negative or positive, depending on the domain (A', B').

Executing Eqs. (32) and (33) in terms of this limited set of atom pairs,

$$\left|\tilde{F}^{\pm}_{\delta}(\mathbf{G}_0)\right|^2 = 4\left[1 - \cos(\pi K_0)\right] \cdot \left[1 + \cos(\pi L_0)\cos\left(-\frac{2\pi b_0}{b}\,K_0 \pm \delta\right)\right] \tag{40}$$

The total scattering intensity for two equally populated domains of the δ angle is hence zero for $K_0 =$ even and, for $K_0 =$odd,

$$I^M_{\mathrm{cal}} = \Phi\,(2.7\,f_{\mathrm{mag}}(Q))^2\left(\mathbf{X}^2_{\perp} + \mathbf{Y}^2_{\perp}\right)\left(N\frac{(2\pi)^3}{\nu_0}\right)\cdot\frac{1}{2}\left[\left|\tilde{F}^{\pm}_{+\delta}(\mathbf{G}_0)\right|^2 + \left|\tilde{F}^{\pm}_{-\delta}(\mathbf{G}_0)\right|^2\right] \tag{41}$$

$$\left[\ldots\right] = 8\left(1 + \cos(\pi L_0)\cdot\cos\left(\frac{2\pi b_0}{b}K_0\right)\cdot\cos(\pm\delta)\right) \tag{42}$$

independent of whether we are looking at the left / right reflection; i.e., the $\pm$ sign in $\tilde{F}_{\pm}$ is completely cancelled out. It is, finally, possible to simplify the magnetic moment projection as

$$\mathbf{X}^2_{\perp} = \mathbf{X}^2 - \left(\mathbf{X}\cdot\hat{\mathbf{Q}}\right)^2 \tag{43}$$

$$\left(\mathbf{X}^2_{\perp} + \mathbf{Y}^2_{\perp}\right)/X^2 = \sin^2\beta + \gamma^2\cos^2\beta \tag{44}$$

with $\gamma^2 = Y^2/X^2$, $\beta = \angle(\hat{\mathbf{Q}}, \mathbf{e}_c)$, and we set $\mathbf{e}_X = \mathbf{e}_c$, $\mathbf{e}_Y = \mathbf{e}_b$.

C. Example: intensity ratio of two reflections

Specifically for two reflections $\mathbf{Q}_1$ and $\mathbf{Q}_2$ in reciprocal space, with Miller indices $(0, 9, 0.207)$ and $(0, 1, 1.207)$, the corresponding Miller indices for the $\mathbf{G}_0$ positions are (090) and (011), respectively. According to Eq. (41) with $\delta = 79°$ from the refinement in Section VI, the intensity ratio is

$$r = \frac{I^M_{\rm cal}(\mathbf{Q}_1)}{I^M_{\rm cal}(\mathbf{Q}_2)} = \frac{\mathcal{L}_1}{\mathcal{L}_2} \cdot \frac{\sin^2\beta_1 + \gamma^2\cos^2\beta_1}{\sin^2\beta_2 + \gamma^2\cos^2\beta_2} \cdot \frac{f_{\rm mag}(Q_1)^2}{f_{\rm mag}(Q_2)^2} \cdot \frac{1 + \cos\left(18\pi\frac{b_0}{b}\right)\cos\delta}{1 - \cos\delta} \tag{45}$$

where $\mathcal{L} = 1/\sin(2\theta)$ (scattering angle 2θ) is the Lorentz factor, which corrects for the scattering geometry. Table II shows the numerical values for various steps in the calculation. From the observed ratio of peak intensities in Fig. 2 of the main text, $r = 0.77$ and $\gamma = Y/X \approx 0.97$ for these two reflections measured on sample A. This γ is somewhat larger than the result for sample B with full refinement in Section VI, but sample A is a larger crystal, with anisotropic shape, where absorption correction is not applied. In particular, the observed intensities are expected to be larger along $(0, 1, L)$, close to transmission geometry. Such limitations of the data quality for sample A do not affect polarization analysis and the qualitative evolution of line scan intensities with temperature.

TABLE II. **Calculation of expected intensity ratio of scattered neutron intensity at** $(0, 9, 0.207)$ **and** $(0, 1, 1.207)$**, based on the magnetic structure model.** The intensity ratio r is calculated from Eq. (45), using these parameters. For convenience, we use $\mathcal{L} = 1/\sin(2\theta)$ (neutron wavelength $\lambda = 1$) in the calculation of the Lorentz factor.

H	K (r.l.u.)	L	Q_a	Q_b ($Å^{-1}$)	Q_c	$\lvert Q\rvert$	2θ (deg.)	$\mathcal{L}(\theta)$	$f_{\rm mag}$	β (deg.)
0	9	0.207	0	2.228	0.302	2.248	51.853	1.272	0.825	82.27
0	1	1.207	0	0.248	1.763	1.780	40.505	1.533	0.885	7.99

D. Structure factor for counter-propagating cycloids

Previously, a stack of counter-propagating helices was reported in γ-Li_2IrO_3 and ascribed to the presence of Kitaev-type anisotropic exchange interactions [31]. Analogously, we consider the structure factor for the helicity stack $\mathbf{h} = (+1, -1, +1, -1)$ as defined in Section IV A. In Eq. (30), $\delta = \pm\pi L_{\mathrm{cyc}}$ ($\delta = \pi \pm \pi L_{\mathrm{cyc}}$) can describe counter-propagating cycloids where the textures of two layers $d = 1, 2$ meet, at certain points, to create pairs of moments that are parallel to the b-axis (parallel to the c-axis). Recognizing that

$$\begin{aligned}\mathbf{m}_\perp &= \mathbf{X}_\perp \cos(h_d \mathbf{q}' \cdot \mathbf{r}_j + \varphi_d) + \mathbf{Y}_\perp \sin(h_d \mathbf{q}' \cdot \mathbf{r}_j + \varphi_d) = \\ &= (\mathbf{X}_\perp + i h_d \mathbf{Y}_\perp) \exp(i\mathbf{q}' \cdot \mathbf{r}_j + i h_d \varphi_d) + \\ &+ (\mathbf{X}_\perp - i h_d \mathbf{Y}_\perp) \exp(-i\mathbf{q}' \cdot \mathbf{r}_j - i h_d \varphi_d)\,,\end{aligned} \tag{46}$$

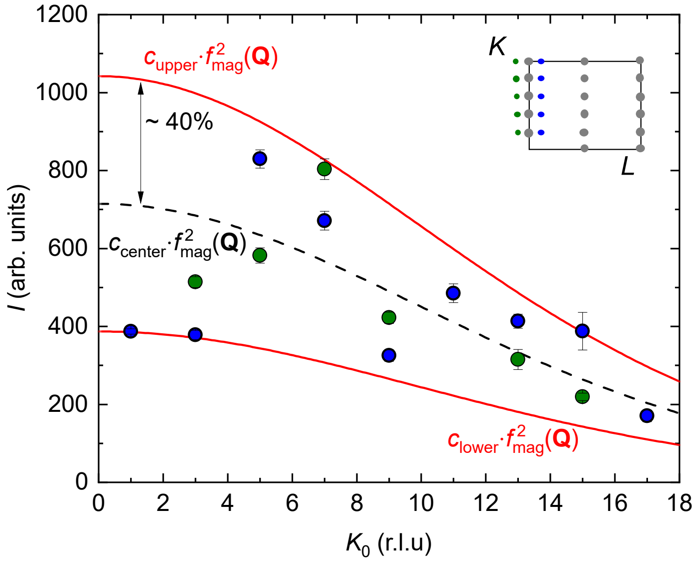

FIG. 6. **Testing analytic model for the magnetic structure factor of the magnetic ground state of $DyTe_3$.** Observed magnetic structure factor, after correction for the Lorentz factor, for incommensurate reflections with $L_0 = 0$. The inset depicts the relevant region of momentum space in the $(0KL)$ scattering plane. The red curves (black dashed line) are envelopes to the data (is the center of the envelopes), bounding the data which oscillates according to Eq. (49). Blue and green are reflections with $\pm\mathbf{q}'$, respectively. Error bars correspond to statistical uncertainties of Gaussian fits to the magnetic reflections. The oscillation amplitude rules out $|\cos\delta| > 0.4$, as discussed in Section IV D.

algebra in line with Section IV B, for one domain of the δ-angle, yields

$$I^M_{\mathrm{cal},\pm} = \Phi\,(2.7\,f_{\mathrm{mag}}(Q))^2 \left(N\frac{(2\pi)^3}{\nu_0}\right)\cdot 4\,[1-\cos(\pi K_0)]\,\cdot$$
$$\cdot\,\left[\mathbf{X}_\perp^2+\mathbf{Y}_\perp^2+\left(\mathbf{X}_\perp^2-\mathbf{Y}_\perp^2\right)\cos\tau \mp 2\mathbf{X}_\perp\cdot\mathbf{Y}_\perp\cdot\sin\tau\right] \tag{47}$$

$$\tau = \frac{2\pi b_0}{b}K_0 - \pi L_0 \pm \delta \tag{48}$$

Special cases can be considered. When $\mathbf{Q}$ is (nearly) parallel to $\mathbf{e}_b$, $\mathbf{Y}_\perp \approx 0$ and the co- and counter-propagating models deliver the same result (now including two δ-domains, introducing a separate $\cos\delta$ factor):

$$I^M_{\mathrm{cal},\pm} \propto (1-\cos(\pi K_0))\;\mathbf{X}_\perp^2\cdot\left(1+\cos(\pi L_0)\cos\delta\cos\left(\frac{2\pi b_0}{b}K_0\right)\right) \tag{49}$$

This is expected from Eq. (46), where additional h_d's appear only before $\mathbf{Y}_\perp$. Figure 6 shows the intensities for $L_0 = 0$, as a function of K_0, with an upper and lower envelope function defined by the magnetic form factor. The maximum amplitude of the oscillation with K, $\sim 40\,\%$, suggests that $|\cos\delta| \leq 0.4$, excluding $\delta = \pm\pi L_{\mathrm{cyc}} = \pm 38^\circ$ and $\delta = \pi \pm 38^\circ$ corresponding to counter-propagating cycloids with high symmetry (see above).

V. ANALYSIS OF NUCLEAR ELASTIC NEUTRON SCATTERING

For sample B, we collected 20 nuclear reflections, which contain 17 independent reflections. Observed and calculated nuclear structure factors are compared to determine the scale factor, a parameter for the extinction correction, and isotropic atomic displacement factors B_{iso}. These three parameters are later used in the magnetic structure analysis (Section VI). For simplicity, we assumed that all the tellurium sites have the same B_{iso}. We performed a least-squares fit and found that the observed and calculated structure factors agree well; $R(F)$ is 3.28 %. The values of B_{iso} for Dy and Te are determined to be 0.1(2) and 0.8(2), respectively. We also performed the same analysis after averaging the structure factors of the equivalent reflections, and obtained the internal $R(F)$ value of 3.35 %.

For sample A, we collected 9 independent nuclear reflections. Observed and calculated nuclear structure factors are compared to determine the scale factor, while fractional coordinates of the atoms and B_{iso} are fixed at the values reported in Ref. [32]. We performed a least-squares fit and found that the observed and calculated structure factors agree well: $R(F) = 7.4\,\%$ (Fig. 8).

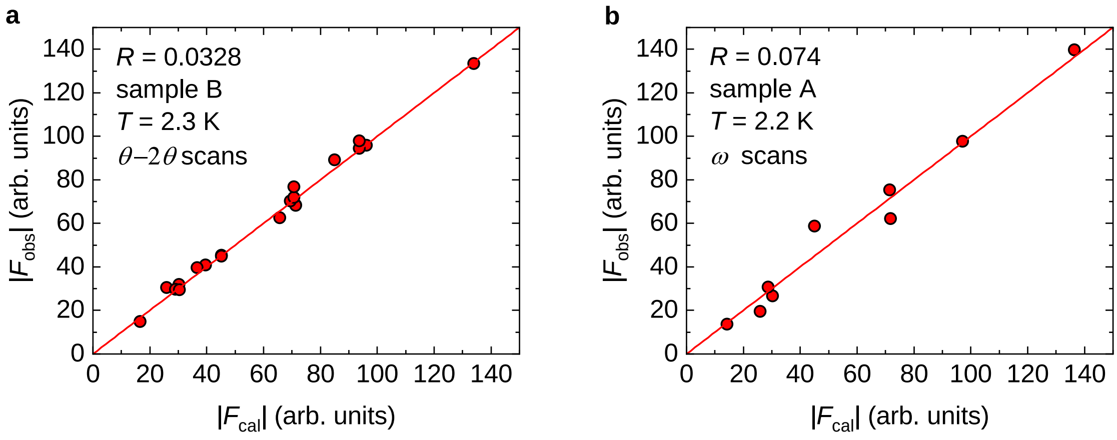

FIG. 7. **Nuclear reflections observed in neutron scattering, consistent with orthorhombic crystal structure of $DyTe_3$.** The observed structure factor F_{obs} is compared to model calculations, F_{cal}, in space group $Cmcm$ as detailed in Section II. We use the reliability index for the structure factor, $R = \sum |F_{\mathrm{obs}} - F_{\mathrm{cal}}| / \sum |F_{\mathrm{obs}}|$, where the sums are over all data points; $R = 0.0328$ for sample B (**a**) and $R = 0.074$ for sample A (**b**) indicate good agreement of model and fit. Note that ω-scans without absorption correction are used to obtain the integrated intensities for sample A.

VI. RESULTS OF MAGNETIC STRUCTURE ANALYSIS

For sample B, we collected 38 (70) Bragg reflections for the commensurate (incommensurate) magnetic reflections, all of which are independent. The polarized neutron scattering experiment in Fig. 12 reveals that the magnetic moments corresponding to the commensurate component are parallel to the a-axis. Assuming the volume fractions of two domains in Fig. 3 to be equal, we performed a least-squares fit to the nonpolarized neutron scattering data from sample B and found that the magnitude of the commensurate component is $5.52(2)\,\mu_B$, with $R(F) = 5.78\,\%$.

For the incommensurate magnetic component, polarized neutron scattering shows that the magnetic moments are confined in the bc-plane. Further considering the existence of the third higher-harmonic magnetic reflections, we assume an elliptic (distorted) cycloidal magnetic modulation, with principal axes parallel to the b- and c-axes, and moment amplitudes m_b and m_c common to all four Dy atoms in a chemical unit cell. We assume equal volume fraction of two domains for the phase shift δ, described in Section IV, and refine m_b,

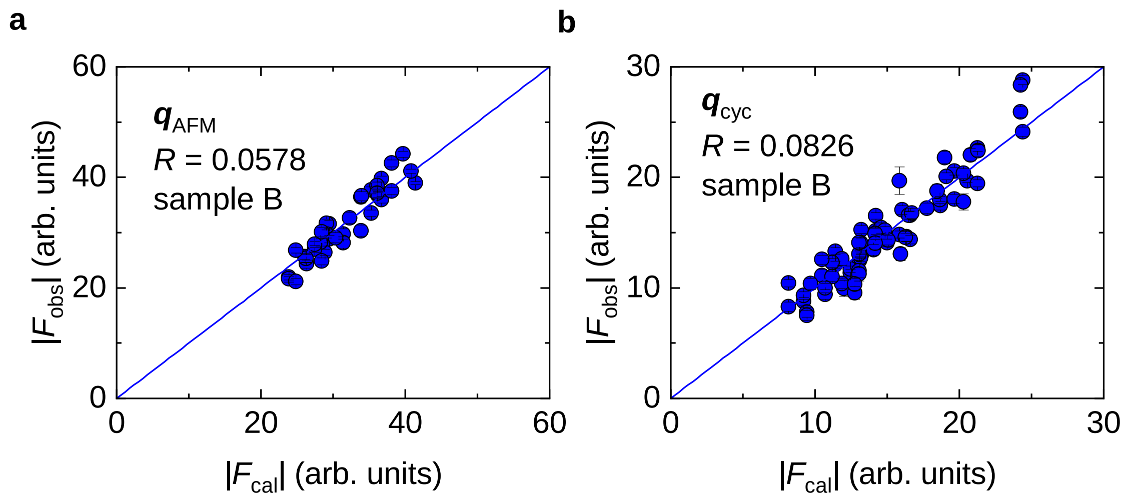

FIG. 8. **Magnetic structure refinement for the ground state of $DyTe_3$.** Antiferromagnetic (commensurate, $\mathbf{q}_{\rm AFM}$) and cycloidal (incommensurate $\mathbf{q}_{\rm cyc}$) components are refined separately from neutron scattering data, without polarization analyzer, at $T = 2.2$ K of sample B. **a**, Averaging two AFM domains with equal weight, we find good agreement [reliability factor $R = 0.0578$] between the measured neutron structure factor $F_{\rm obs}$ and model calculations $F_{\rm cal}$. **b**, Likewise, good agreement is found when summing two δ-domains for the incommensurate order $\mathbf{q}_{cyc}$. Here, we minimized R by adjusting the value of the phase shift δ between sheets in a bilayer, as defined in Fig. 3 **g** of the main text, and the elliptic distortion Y/X of the cycloid (Section VI). The error bars in both panels correspond to statistical uncertainties of Gaussian fits to the integrated neutron scattering intensity.

m_c, and δ by least-squares analysis. This yields $3.93(3)\,\mu_B$, $6.61(3)\,\mu_B$, and $\pm 79.2(5)$ deg, respectively. The $R(F)$ value is 8.26 % (Fig. 8) and combined, the total moment length at the Dy site is

$$m_{\rm tot} = \sqrt{m_a^2 + \max\left(m_b^2, m_c^2\right)} = 8.61 \pm 0.1\,\mu_{\rm B}/\mathrm{Dy} \tag{50}$$

at $T = 2.2$ K, which is about $T/T_{\rm N} = 0.6$, i.e. still rather close to the critical temperature; the ordered moment at absolute zero temperature is expected to be close to the local-ion value of $10\,\mu_{\rm B}$ per dysprosium, also due to an underestimation in our calculation by neglecting higher harmonics contributions. The difference in value between m_b and m_c is consistent with the presence of third harmonic magnetic reflections; such distortions and higher harmonics have also been observed in insulating multiferroics [33].

Our model demonstrates that, at least, the incommensurate component involves both m_b

and m_c components, of different amplitudes. Further experiments using spherical neutron polarimetry will be suitable to investigate, in more detail, the directions of the minor and major axes for the spin ellipsis in the bc-plane.

The lattice constants $a = c = 4.302\,\text{Å}$, $b = 25.381\,\text{Å}$ are determined from neutron scattering at low temperature. These values are close to the literature values for the orthorhombic, yet nearly tetragonal structure of $DyTe_3$, $a = c = 4.296\,\text{Å}$, $b = 25.450\,\text{Å}$ [32]. Our neutron measurements are not able to pick up minute atomic displacements due to the CDW, which can be seen in careful x-ray studies [26]; we used the fractional atomic positions of the average structure from Ref. [32] in our analysis of the nuclear and magnetic scattering.

VII. ANISOTROPIC ELECTRONIC TRANSPORT PROPERTIES

Angle-resolved photoemission (ARPES) studies combined with a tight binding (TB) model in Ref. [34] reveals that, in the family of the rear-earth tritellurides RTe$_3$, the states at the Fermi level are mainly formed by the in-plane p_x and p_z orbitals of the Te-A ions in the Te square net, c.f. Fig. 1 **a** of the main text. Indeed, they are well separated by more than 1 eV from other bands, already indicating anisotropic bonding and transport properties. With a standard four-probe method and a modified Montgomery geometry Ru *et al.* determined both in-plane (ρ_{ac}) and out-of-plane resistance (ρ_b) of various RTe$_3$ compounds [35]. They differ by at least one order of magnitude ($\rho_b \gg \rho_{ac}$), consistent with a metallic Te square net as well as covalently bonded RTe slabs.

To further extract the in-plane anisotropy ratio ρ_c/ρ_a of $DyTe_3$, we use the Montgomery technique and follow the method described in Ref. [36]. From temperature dependent resistance measurements $R_a(T)$ and $R_c(T)$, we calculate the shape of a hypothetical isotropic sample with dimensions $L_a \times L_b \times L_c$ and same absolute resistance values R'_a and R'_c

$$L = \frac{L_c}{L_a} = \frac{1}{2}\left[\frac{1}{\pi}\ln\left(\frac{R_c}{R_a}\right) + \sqrt{\left(\frac{1}{\pi}\ln\left(\frac{R_c}{R_a}\right)\right)^2 + 4}\right] \tag{51}$$

Based on published data from Ref. [35] and our sample geometry, a thin exfoliated flake, we estimated that the calculation of the anisotropy ratio ρ_c/ρ_a is possible in the thin-layer

limit. Following Ref. [36] and using the real sample dimensions $l_a \times l_b \times l_c$ this leads to

$$\rho_a = \frac{\pi}{8}\,\frac{l_b\,l_c}{l_a}\,L^{-1}\,R_a\,\sinh\left(\pi L\right) \tag{52}$$

$$\rho_c = \frac{\pi}{8}\,\frac{l_b\,l_a}{l_c}\,L\,R_a\,\sinh\left(\pi L\right) \tag{53}$$

and finally to

$$\frac{\rho_c}{\rho_a} = \left(\frac{l_a}{l_c}\,L\right)^2 \tag{54}$$

There is some ambiguity in the interpretation of resistance anisotropy changes at T_N, which can be ascribed either to opening of a partial charge gap in the ordered state, or to fluctuations in the paramagnetic regime and their suppression below T_N. In $DyTe_3$, the sign of the observed change in ρ_c/ρ_a implies that, when moving from the paramagnetic into the ordered regime, the resistance along the c-axis becomes larger than the resistance along the a-axis. This result can be neatly explained by attributing partial gap opening to $\mathbf{q}_{\mathrm{AFM}} = (0, b^*, 0.5c^*)$, $\mathbf{q}_{\mathrm{cyc}} = (0, b^*, 0.207c^*)$, while unidirectional fluctuations should enhance ρ_c/ρ_a above T_N, and suppress it below T_N.

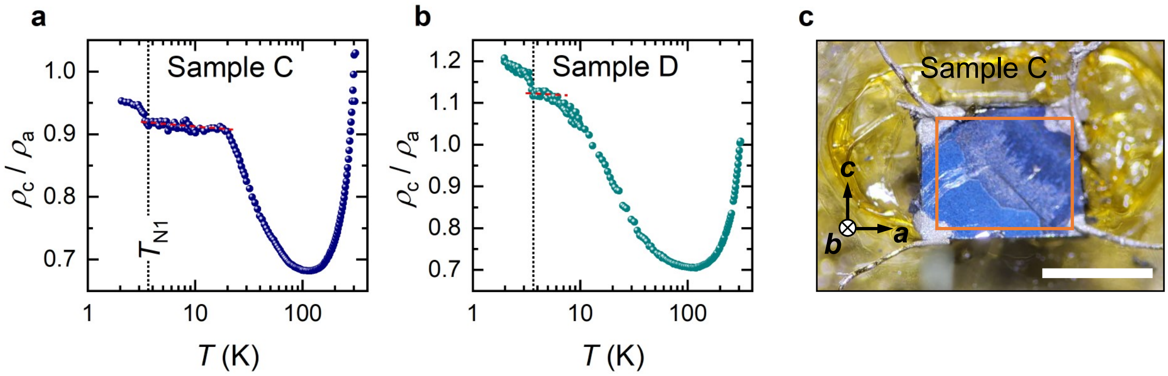

FIG. 9. **Anisotropy of electrical transport properties in ac basal plane of $DyTe_3$. a,b**, ac basal plane resistance ratio ρ_c/ρ_a as a function of temperature for different samples. In Sample D, the assumed rectangular sample shape (c.f. panel **c**) is slightly – in particular 4 % along the a direction – adjusted to match $\rho_c/\rho_a \sim 1$ at $T > T_{\mathrm{CDW}}$, c.f. Eq. (54). Both samples show a clear kink at the magnetic phase transition T_{N1}, followed by a broad plateau region, indicated by a red dashed line, with nearly constant ratio. **c**, Sample C mounted in Montgomery geometry. The orange rectangle depicts the assumed sample shape that is used to estimate the length ratio $l_a/l_c = 1.266$, where the white scale bar on the bottom right corresponds to 1 mm.

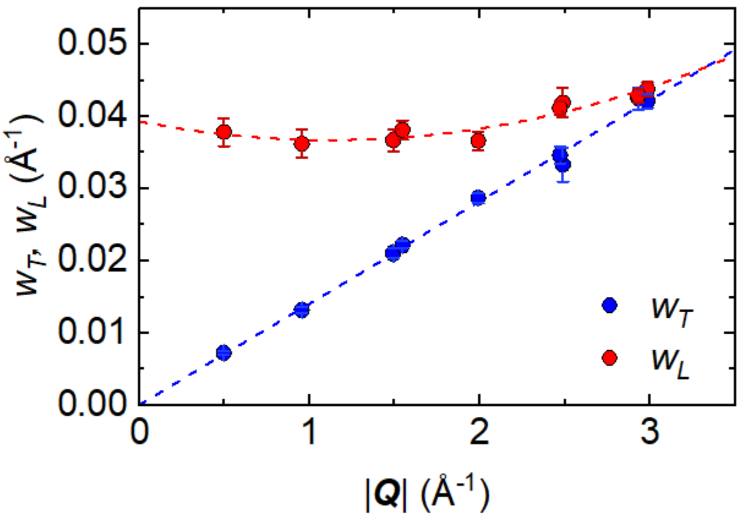

FIG. 10. **Calibration of instrument resolution at 5G-PONTA at the JRR-3 neutron reactor source, for sample A.** Each pair of (red, blue) data points corresponds to the full width at half maximum (FWHM) of a nuclear lattice reflection. The shape of the resolution ellipsoid in the b^*-c^* plane is defined by the width of an ω-scan (w_T, transverse width) and the width of a $\omega - 2\theta$ scan (w_L, longitudinal width), respectively. These parameters depend on the sample shape, crystal quality, the momentum transfer $|\mathbf{Q}|$, and the performance of the instrument. Blue and red dashed lines are a linear fit, and a second order polynomial fit to the data, respectively. These fits can be used for estimation of the instrument resolution at arbitrary positions in the bc scattering plane, e.g. in Fig. 4 **c** of the main text. Error bars correspond to statistical uncertainties of Gaussian fits to the nuclear reflections.

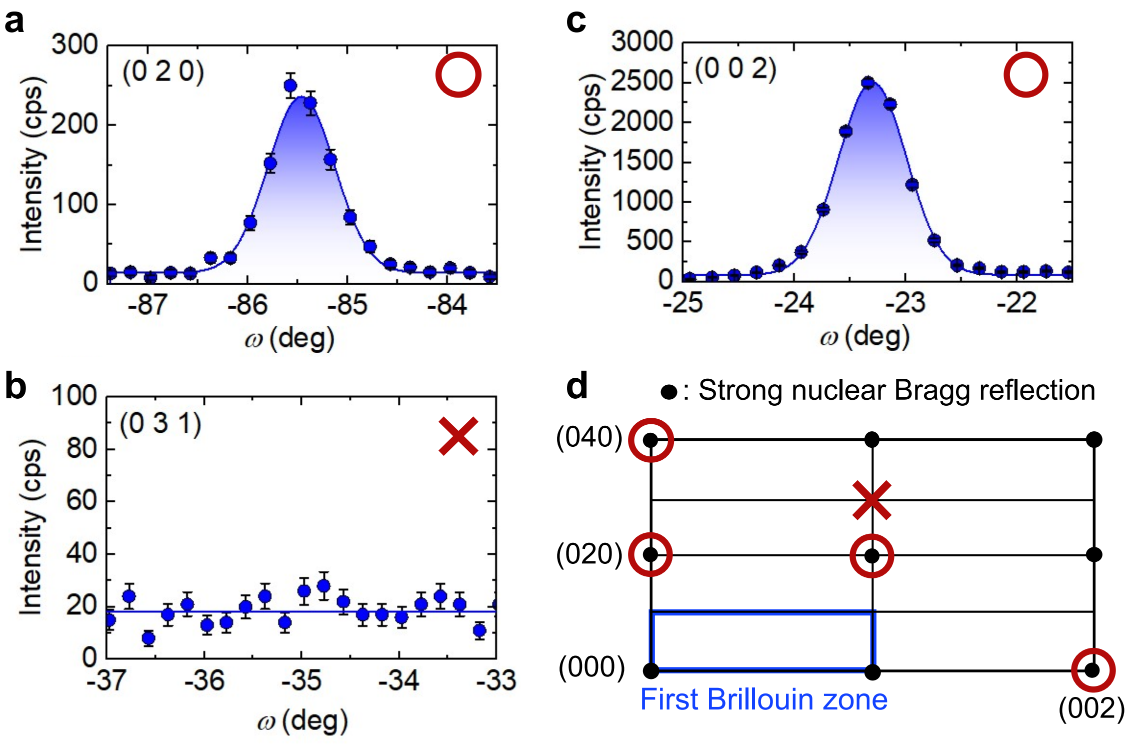

FIG. 11. **Confirmation of crystallographic extinction rule in $DyTe_3$ in sample A.** Reflections $h + k$ = odd are forbidden in $Cmcm$ (space group 63), or specifically $k = 2n$ is required in the $(0KL)$ scattering plane, which is used for the present experiment. We observe zero intensity at (031) and 800 counts per second (cps) at (021); the latter reflection is not shown in this figure. When instead rotating the sample into the $(HK0)$ scattering plane, (130) has 1200 cps and (120) exhibits zero intensity. Specifically, the comparison of (130) and (031) allows us to confirm the alignment of the crystal in our neutron experiment. The error bars correspond to Poisson counting errors of the integrated neutron scattering intensity.

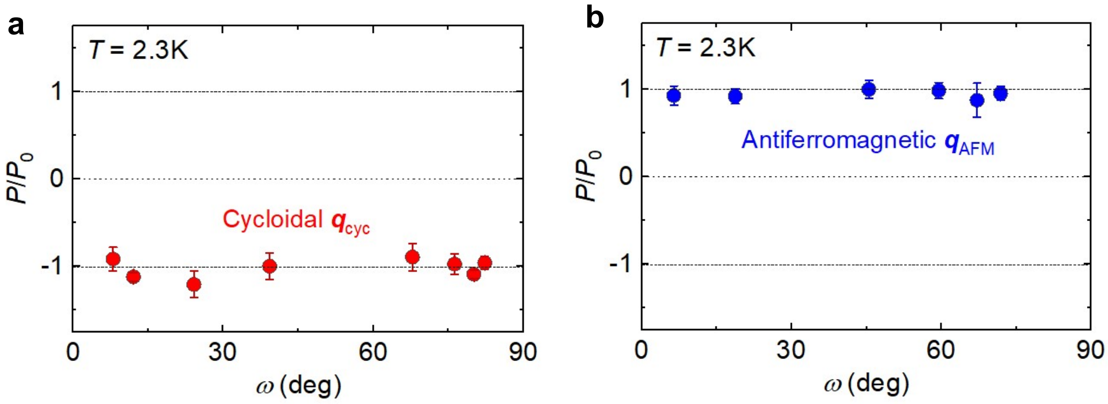

FIG. 12. **Neutron flipping ratio in sample A for two components of the magnetic order in $DyTe_3$'s ground state.** Here, the flipping ratio $P = (I_{\rm NSF} - I_{\rm SF})/(I_{\rm NSF} + I_{\rm SF})$ is the normalized ratio of spin flip (SF) and non-spin flip (NSF) intensities for a magnetic reflection. P_0 is the flipping ratio at the nuclear reflection (002) (Methods). In our geometry, SF intensity is dominated by the b-component (the c-component) of the magnetization at $\omega \approx 0°$ (at $\omega \approx 90°$), although the structure and magnetic form factors have to be carefully taken into account when comparing intensities of various reflections (Section VI). **a**, The incommensurate magnetization component at $\mathbf{q}_{\rm cyc}$ gives dominant SF scattering, with $P/P_0 \equiv -1$ independent of the ω angle. This implies presence of both m_b and m_c for the incommensurate reflection. **b**, The antiferromagnetic reflection $\mathbf{q}_{\rm AFM}$ has dominant NSF scattering, which is again independent of ω and consistent with magnetization component exclusively along the a-axis; hence leading to $P/P_0 \equiv +1$ for all reflections measured. Error bars in both panels are derived by error propagation of the corresponding statistical uncertainties of Gaussian fits to the neutron scattering intensity of the respective peak.

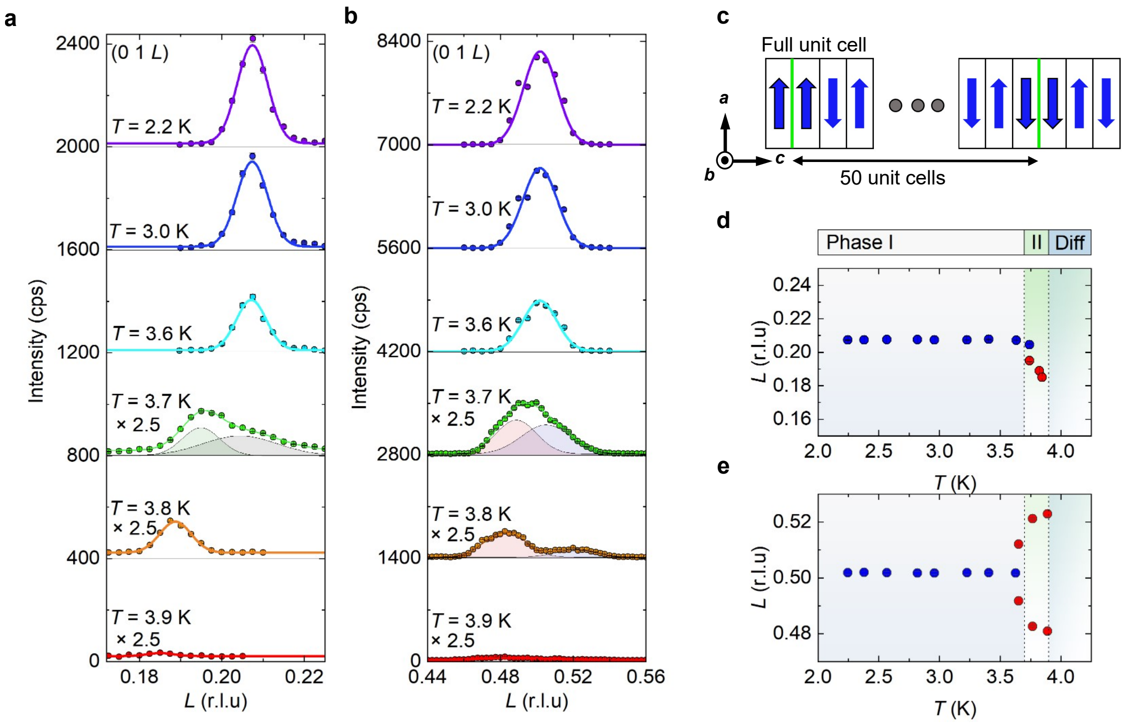

FIG. 13. **Incommensurate and commensurate antiferromagnetic component in phase II of $DyTe_3$ (sample A).** **a**,**b**, Line scans of magnetic intensity through the incommensurate (**a**) and commensurate (**b**) reflection on the $(01L)$ line, with clear temperature dependence. Shaded Gaussian curves at $T = 3.7\,\mathrm{K}$ indicate a double-Gaussian fit in the regime of phase coexistence between phases I and II. High temperature data is multiplied by a scale factor to enhance visibility. The magnetic intensity vanishes in the incommensurate line entirely at $T_{\mathrm{N2}} = 3.85\,\mathrm{K}$, with no indications of diffuse scattering in the thermally disordered regime. Above T_{N2}, we observe a weak, diffuse neutron signal along the $(0, K, 1/2)$ line, with rapid decay of the coherence length. The error bars correspond to Poisson counting errors of the integrated neutron scattering intensity. **c**, Illustration of discommensuration-driven shift of q_{AFM} in $\mathbf{q}_{\mathrm{AFM}} = (0, b^*, q_{\mathrm{AFM}})$ for phase II, where a defect every ~ 50 unit cells is introduced to release magnetoelastic strain built up between the crystal lattice and the collinear antiferromagnetic structure. **d**,**e**, Temperature dependence of $\mathbf{q}_{\mathrm{cyc}}$ and $\mathbf{q}_{\mathrm{AFM}}$, respectively. The different regimes, phase I, II and the diffuse scattering regime (Diff), are highlighted by different color shadings. Error bars correspond to statistical uncertainties of Gaussian fits to the magnetic reflections.

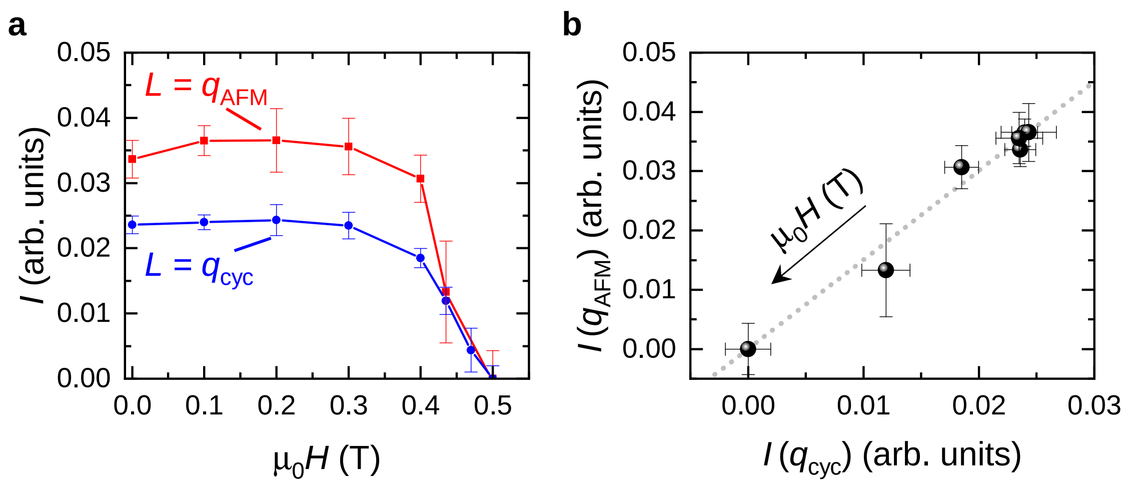

FIG. 14. **Coupling of commensurate and incommensurate magnetic order in $DyTe_3$, tracked by small-angle neutron scattering (SANS) in a magnetic field along the c-axis, for sample E. a**, Integrated intensity of commensurate ($\mathbf{q} = \mathbf{q}_{\mathrm{AFM}}$) and incommensurate ($\mathbf{q} = \mathbf{q}_{\mathrm{cyc}}$) reflections of type $(0, -1, -L)$ as a function of magnetic field and at a temperature of $T = 2\,\mathrm{K}$. The observed intensity of both drops simultaneously at around $\mu_0 H = 0.4\,\mathrm{T}$, with a fixed intensity ratio $I(\mathbf{q}_{\mathrm{AFM}})/I(\mathbf{q}_{\mathrm{cyc}}) \sim 1.5$, as indicated by a grey dashed line in panel **b**. Error bars correspond to statistical uncertainties of Gaussian fits to the extracted linecuts, c.f. panels **c** and **e** in Fig. 5 of the main text.

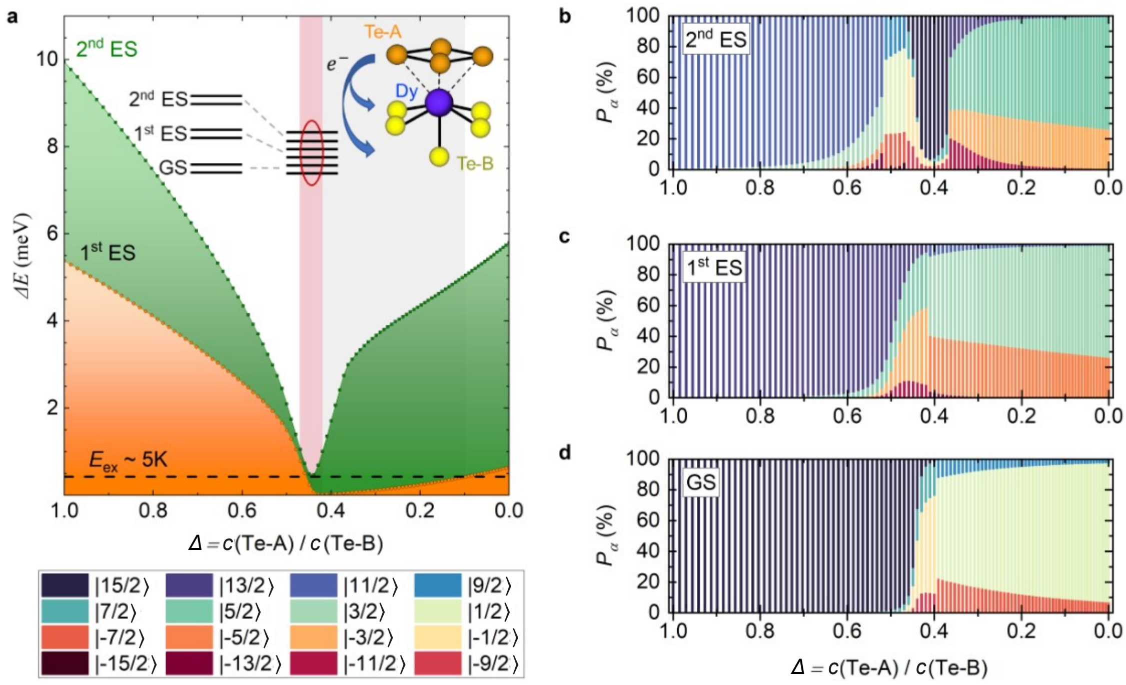

FIG. 15. **Crystal electric field calculations and local environment of Dy in $DyTe_3$. a**, Evolution of the energy gap ΔE_i between ground state doublet (GS, $|\psi_0^\pm\rangle$) and the first (1st ES, $|\psi_1^\pm\rangle$) or second (2nd ES, $|\psi_2^\pm\rangle$) excited state Kramers doublets. We consider a (virtual) charge transfer from the metallic Te_2 square net (Te-A) to the covalent bonded Tellurium (Te-B) ions, as sketched in the inset. The x-axis is labeled by the ratio of effective point charges c on the two types of Te ions. Within the red (grey) shaded area, the excitation gaps to the the first and second excited states are (to the first excited state is) on the order of the exchange interaction energy $E_{\text{ex}} \sim 2\,\text{K}$ (dashed horizontal line). Panels **b-d** show the composition of $\left|\psi_{0,1,2}^+\right\rangle$ in terms of eigenstates of (b-component of) total angular momentum $|J_b = \pm n/2\rangle$ with $n \leq 15$, as suitable for the $4f^9$ shell of dysprosium. The P_α are probabilities, i.e. absolute squares of amplitudes, for each contribution to the total wavefunction.

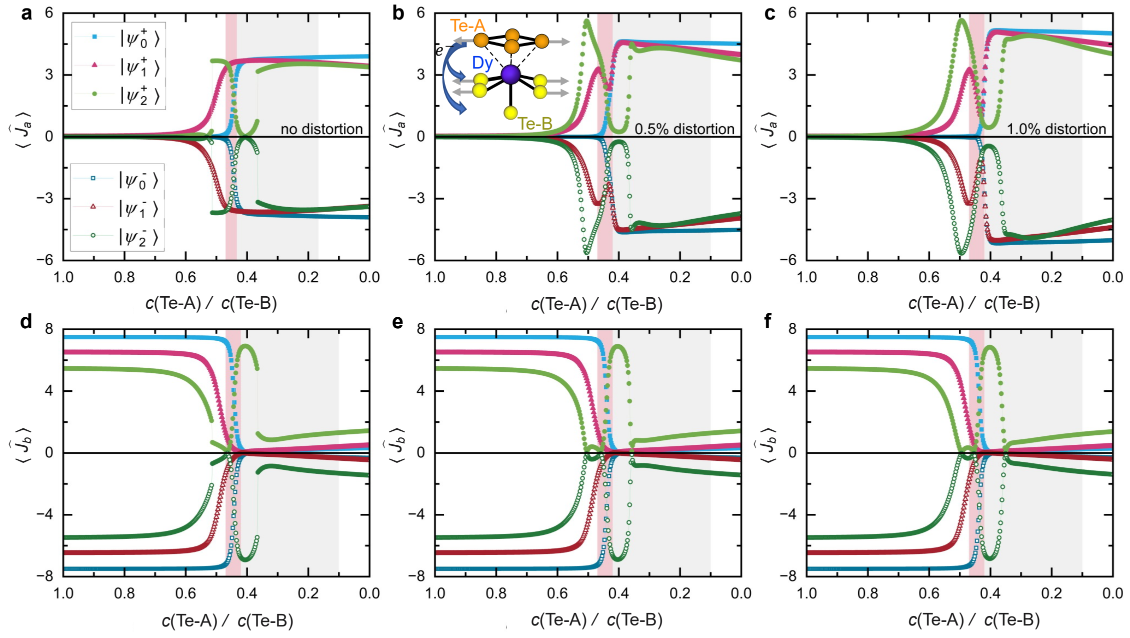

FIG. 16. **Mixing of crystal field states in $DyTe_3$ by exchange interactions. a-c**, The maximal and minimal eigenvalues of $\hat{J}_a$ and **d-f** of $\hat{J}_b$ calculated for the crystal field doublets $\left|\psi^{\pm}_{0,1,2}\right\rangle$. The charge ratio ($x$-coordinate) describes the ratio of effective charges assigned to Te-A and Te-B tellurium ions corresponding to metallic and ionic bonds around Dy, respectively. A matrix representation of the operators $\hat{J}_a$ and $\hat{J}_b$ is calculated on each respective subspace spanned by a Kramers pair of states, and subsequently diagonalized. There is a transition from dominant $\left\langle\psi_0\left|\hat{J}_b\right|\psi_0\right\rangle = \pm 1/2$ (easy-plane) to $\pm 15/2$ (easy-axis) for the ground state $|\psi_0\rangle$, when reducing the effective crystal electric field charge for Te-A on metallic bonds. The first excited state behaves similarly, while the second excited state generally covers a broader range of $\hat{J}_b$ eigenvalues. Red and grey shaded areas are defined as in Fig. 15. In the red region, the character of the ground state (GS, $|\psi_0^{\pm}\rangle$, left column) changes from predominant $|J_b = \pm 15/2\rangle$ to mainly $|J_b = \pm 1/2\rangle$ character. It is not possible to generate sizable in-plane (b-axis) magnetic moment using any linear combination of $|\psi_0^+\rangle$, $|\psi_0^-\rangle$ at relative weight of CEF charges c on Te-A and Te-B sites > 0.5 (< 0.4).

[1] Y. Gao, Q. Yin, Q. Wang, Z. Li, J. Cai, T. Zhao, H. Lei, S. Wang, Y. Zhang, and B. Shen, Spontaneous (Anti)meron Chains in the Domain Walls of van der Waals Ferromagnetic $Fe_{5-x}GeTe_2$, Advanced Materials **32**, 2005228 (2020).

[2] T. T. Ly, J. Park, K. Kim, H.-B. Ahn, N. J. Lee, K. Kim, T.-E. Park, G. Duvjir, N. H. Lam, K. Jang, C.-Y. You, Y. Jo, S. K. Kim, C. Lee, S. Kim, and J. Kim, Direct Observation of Fe-Ge Ordering in $Fe_{5-x}GeTe_2$ Crystals and Resultant Helimagnetism, Advanced Functional Materials **31**, 2009758 (2021).

[3] A. F. May, C. A. Bridges, and M. A. McGuire, Physical properties and thermal stability of $Fe_{5-x}GeTe_2$ single crystals, Physical Review Materials **3**, 104401 (2019).

[4] M. Baenitz, M. M. Piva, S. Luther, J. Sichelschmidt, K. M. Ranjith, H. Dawczak-Dębicki, M. O. Ajeesh, S.-J. Kim, G. Siemann, C. Bigi, P. Manuel, D. Khalyavin, D. A. Sokolov, P. Mokhtari, H. Zhang, H. Yasuoka, P. D. C. King, G. Vinai, V. Polewczyk, P. Torelli, J. Wosnitza, U. Burkhardt, B. Schmidt, H. Rosner, S. Wirth, H. Kühne, M. Nicklas, and M. Schmidt, Planar triangular $S = 3/2$ magnet $AgCrSe_2$: Magnetic frustration, short range correlations, and field-tuned anisotropic cycloidal magnetic order, Physical Review B **104**, 134410 (2021).

[5] U. K. Gautam, R. Seshadri, S. Vasudevan, and A. Maignan, Magnetic and transport properties, and electronic structure of the layered chalcogenide $AgCrSe_2$, Solid state communications **122**, 607 (2002).

[6] T. Kurumaji, S. Seki, S. Ishiwata, H. Murakawa, Y. Kaneko, and Y. Tokura, Magnetoelectric responses induced by domain rearrangement and spin structural change in triangular-lattice helimagnets NiI_2 and CoI_2, Physical Review B **87**, 014429 (2013).

[7] D. Lebedev, J. T. Gish, E. S. Garvey, T. K. Stanev, J. Choi, L. Georgopoulos, T. W. Song, H. Y. Park, K. Watanabe, T. Taniguchi, N. P. Stern, V. K. Sangwan, and M. C. Hersam, Electrical Interrogation of Thickness-Dependent Multiferroic Phase Transitions in the 2D Antiferromagnetic Semiconductor NiI_2, Advanced Functional Materials **33**, 2212568 (2023).

[8] A. Adam, D. Billerey, C. Terrier, R. Mainard, L. Regnault, J. Rossat-Mignod, and P. Mériel, Neutron diffraction study of the commensurate and incommensurate magnetic structures of $NiBr_2$, Solid State Communications **35**, 1 (1980).

[9] Y. Tokunaga, D. Okuyama, T. Kurumaji, T. Arima, H. Nakao, Y. Murakami, Y. Taguchi, and Y. Tokura, Multiferroicity in $NiBr_2$ with long-wavelength cycloidal spin structure on a triangular lattice, Physical Review B **84**, 060406 (2011).

[10] C. R. Ronda, G. J. Arends, and C. Haas, Photoconductivity of the nickel dihalides and the nature of the energy gap, Physical Review B **35**, 4038 (1987).

[11] T. Kurumaji, S. Seki, S. Ishiwata, H. Murakawa, Y. Tokunaga, Y. Kaneko, and Y. Tokura, Magnetic-Field Induced Competition of Two Multiferroic Orders in a Triangular-Lattice Helimagnet MnI_2, Physical Review Letters **106**, 167206 (2011).

[12] N. J. Ghimire, M. A. McGuire, D. S. Parker, B. Sipos, S. Tang, J.-Q. Yan, B. C. Sales, and D. Mandrus, Magnetic phase transition in single crystals of the chiral helimagnet $Cr_{1/3}NbS_2$, Physical Review B **87**, 104403 (2013).

[13] K. Lu, A. Murzabekova, S. Shim, J. Park, S. Kim, L. Kish, Y. Wu, L. DeBeer-Schmitt, A. A. Aczel, A. Schleife, N. Mason, F. Mahmood, and G. J. MacDougall, Understanding the Anomalous Hall effect in $Co_{1/3}NbS_2$ from crystal and magnetic structures (2022), arXiv:2212.14762 [cond-mat.mtrl-sci].

[14] G. Tenasini, E. Martino, N. Ubrig, N. J. Ghimire, H. Berger, O. Zaharko, F. Wu, J. F. Mitchell, I. Martin, L. Forró, and A. F. Morpurgo, Giant anomalous Hall effect in quasi-two-dimensional layered antiferromagnet $Co_{1/3}NbS_2$, Physical Review Research **2**, 023051 (2020).

[15] H. Takagi, R. Takagi, S. Minami, T. Nomoto, K. Ohishi, M.-T. Suzuki, Y. Yanagi, M. Hirayama, N. Khanh, K. Karube, H. Saito, D. Hashizume, R. Kiyanagi, Y. Tokura, R. Arita, T. Nakajima, and S. Seki, Spontaneous topological Hall effect induced by non-coplanar antiferromagnetic order in intercalated van der Waals materials, Nature Physics **19**, 961 (2023).

[16] Y. Kousaka, T. Ogura, J. Zhang, P. Miao, S. Lee, S. Torii, T. Kamiyama, J. Campo, K. Inoue, and J. Akimitsu, Long Periodic Helimagnetic Ordering in CrM_3S_6 (M = Nb and Ta), Journal of Physics: Conference Series **746**, 012061 (2016).

[17] T. Miyadai, K. Kikuchi, H. Kondo, S. Sakka, M. Arai, and Y. Ishikawa, Magnetic Properties of $Cr_{1/3}NbS_2$, Journal of the Physical Society of Japan **52**, 1394 (1983).

[18] L. Wang, N. Chepiga, D.-K. Ki, L. Li, F. Li, W. Zhu, Y. Kato, O. S. Ovchinnikova, F. Mila, I. Martin, D. Mandrus, and A. F. Morpurgo, Controlling the Topological Sector of Magnetic Solitons in Exfoliated $Cr_{1/3}NbS_2$ Crystals, Physical Revie Letters **118**, 257203 (2017).

[19] D. Obeysekera, K. Gamage, Y. Gao, S.-w. Cheong, and J. Yang, The Magneto-Transport

Properties of $Cr_{1/3}TaS_2$ with Chiral Magnetic Solitons, Advanced Electronic Materials **7**, 2100424 (2021).

[20] C. Zhang, J. Zhang, C. Liu, S. Zhang, Y. Yuan, P. Li, Y. Wen, Z. Jiang, B. Zhou, Y. Lei, D. Zheng, C. Song, Z. Hou, W. Mi, U. Schwingenschlögl, A. Manchon, Z. Q. Qiu, H. N. Alshareef, Y. Peng, and X.-X. Zhang, Chiral Helimagnetism and One-Dimensional Magnetic Solitons in a Cr-Intercalated Transition Metal Dichalcogenide, Advanced Materials **33**, 2101131 (2021).

[21] C.-H. Zhang, H. Algaidi, P. Li, Y. Yuan, and X.-X. Zhang, Magnetic soliton confinement and discretization effects in $Cr_{1/3}TaS_2$ nanoflakes, Rare Metals **41**, 3005 (2022).

[22] C. Malliakas and M. Kanatzidis, Divergence in the Behavior of the Charge Density Wave in $RETe_3$ (RE= Rare-Earth element) with Temperature and RE Element, Journal of the American Chemical Society **128**, 12612 (2006).

[23] Tables of Form Factors - Institut Laue Langevin, Grenoble, France, `https://www.ill.eu/sites/ccsl/ffacts/`, accessed: 2023-03-31.

[24] G. L. Squires, *Introduction to the Theory of Thermal Neutron Scattering*, 3rd ed. (Cambridge University Press, 2012).

[25] V. Slovyanskikh, N. Kuznetsov, and N. Gracheva, The Dy-U-Te system, Russian Journal of Inorganic Chemistry **30**, 1666 (1985).

[26] C. Malliakas, S. J. L. Billinge, H. J. Kim, and M. G. Kanatzidis, Square Nets of Tellurium: Rare-Earth Dependent Variation in the Charge-Density Wave of $RETe_3$ (RE = Rare-Earth Element), Journal of the American Chemical Society **127**, 6510 (2005).

[27] M. I. Aroyo, A. Kirov, C. Capillas, J. M. Perez-Mato, and H. Wondratschek, Bilbao Crystallographic Server II: Representations of crystallographic point groups and space groups, Acta Crystallographica **A62**, 115 (2006).

[28] M. Kenzelmann, A. B. Harris, A. Aharony, O. Entin-Wohlman, T. Yildirim, Q. Huang, S. Park, G. Lawes, C. Broholm, N. Rogado, R. J. Cava, K. H. Kim, G. Jorge, and A. P. Ramirez, Field dependence of magnetic ordering in Kagomé-staircase compound $Ni_3V_2O_8$, Phys. Rev. B **74**, 014429 (2006).

[29] P. Brown, T. Chattopadhyay, J. Forsyth, and V. Nunez, Magnetic phase transitions of $MnWO_4$ studied by the use of neutron diffraction, J. Phys.: Condens. Matter **48**, 4281 (1991).

[30] G. Lautenschläger, H. Weitzel, T. Vogt, R. Hock, A. Böhm, M. Bonnet, and H. Fuess, Magnetic phase transitions of $MnWO_4$ studied by the use of neutron diffraction, Phys. Rev. B **48**, 6087

(1993).

[31] A. Biffin, R. D. Johnson, I. Kimchi, R. Morris, A. Bombardi, J. G. Analytis, A. Vishwanath, and R. Coldea, Noncoplanar and Counterrotating Incommensurate Magnetic Order Stabilized by Kitaev Interactions in $\gamma{-}Li_2IrO_3$, Phys. Rev. Lett. **113**, 197201 (2014).

[32] M. P. Pardo and J. Flahaut, Les tellurures superieurs des elements des terres rares, de formules L_2Te_5 et LTe_3, Bulletin de la Société Chimique de France , 3658 (1967).

[33] M. Kenzelmann, A. B. Harris, S. Jonas, C. Broholm, J. Schefer, S. B. Kim, C. L. Zhang, S.-W. Cheong, O. P. Vajk, and J. W. Lynn, Magnetic Inversion Symmetry Breaking and Ferroelectricity in $TbMnO_3$, Phys. Rev. Lett. **95**, 087206 (2005).

[34] V. Brouet, W. L. Yang, X. J. Zhou, Z. Hussain, R. G. Moore, R. He, D. H. Lu, Z. X. Shen, J. Laverock, S. B. Dugdale, N. Ru, and I. R. Fisher, Angle-resolved photoemission study of the evolution of band structure and charge density wave properties in $R\mathrm{Te}_3$ ($R =$ Y, La, Ce, Sm, Gd, Tb, and Dy), Physical Review B **77**, 235104 (2008).

[35] N. Ru, C. Condron, G. Margulis, K. Shin, J. Laverock, S. Dugdale, M. Toney, and I. Fisher, Effect of chemical pressure on the charge density wave transition in rare-earth tritellurides RTe_3, Physical Review B **77**, 035114 (2008).

[36] C. Dos Santos, A. De Campos, M. Da Luz, B. White, J. Neumeier, B. De Lima, and C. Shigue, Procedure for measuring electrical resistivity of anisotropic materials: A revision of the Montgomery method, Journal of Applied Physics **110** (2011).
